# Hypoxia and TGF-β Drive Breast Cancer Bone Metastases through Parallel Signaling Pathways in Tumor Cells and the Bone Microenvironment

**DOI:** 10.1371/journal.pone.0006896

**Published:** 2009-09-03

**Authors:** Lauren K. Dunn, Khalid S. Mohammad, Pierrick G. J. Fournier, C. Ryan McKenna, Holly W. Davis, Maria Niewolna, Xiang Hong Peng, John M. Chirgwin, Theresa A. Guise

**Affiliations:** 1 Division of Endocrinology, Department of Medicine, University of Virginia, Charlottesville, Virginia, United States of America; 2 Department of Biochemistry and Molecular Genetics, University of Virginia, Charlottesville, Virginia, United States of America; Roswell Park Cancer Institute, United States of America

## Abstract

**Background:**

Most patients with advanced breast cancer develop bone metastases, which cause pain, hypercalcemia, fractures, nerve compression and paralysis. Chemotherapy causes further bone loss, and bone-specific treatments are only palliative. Multiple tumor-secreted factors act on the bone microenvironment to drive a feed-forward cycle of tumor growth. Effective treatment requires inhibiting upstream regulators of groups of prometastatic factors. Two central regulators are hypoxia and transforming growth factor (TGF)- β. We asked whether hypoxia (via HIF-1α) and TGF-β signaling promote bone metastases independently or synergistically, and we tested molecular versus pharmacological inhibition strategies in an animal model.

**Methodology/Principal Findings:**

We analyzed interactions between HIF-1α and TGF-β pathways in MDA-MB-231 breast cancer cells. Only vascular endothelial growth factor (VEGF) and the CXC chemokine receptor 4 (CXCR4), of 16 genes tested, were additively increased by both TGF-β and hypoxia, with effects on the proximal promoters. We inhibited HIF-1α and TGF-β pathways in tumor cells by shRNA and dominant negative receptor approaches. Inhibition of either pathway decreased bone metastasis, with no further effect of double blockade. We tested pharmacologic inhibitors of the pathways, which target both the tumor and the bone microenvironment. Unlike molecular blockade, combined drug treatment decreased bone metastases more than either alone, with effects on bone to decrease osteoclastic bone resorption and increase osteoblast activity, in addition to actions on tumor cells.

**Conclusions/Significance:**

Hypoxia and TGF-β signaling in parallel drive tumor bone metastases and regulate a common set of tumor genes. In contrast, small molecule inhibitors, by acting on both tumor cells and the bone microenvironment, additively decrease tumor burden, while improving skeletal quality. Our studies suggest that inhibitors of HIF-1α and TGF-β may improve treatment of bone metastases and increase survival.

## Introduction

Breast cancers frequently metastasize to bone, where they disrupt normal bone remodeling to cause bone destruction, pain, pathologic fracture, hypercalcemia, and nerve compression [1]. Besides conventional radiation and chemotherapy, bisphosphonates are the only treatment available for patients with bone metastases. These drugs decrease skeletal morbidity and provide palliative relief but no cure [1]. Bone is a unique microenvironment in which breast cancer thrives. Growth factors, such as transforming growth factor-β (TGF- β) are stored in the mineralized bone matrix. Breast cancers that metastasize to bone secrete factors, such as parathyroid hormone-related protein (PTHrP) and interleukin-11 (IL-11), that stimulate osteoclastic bone destruction and the release and activation of growth factors immobilized in the bone matrix. These factors in turn act on tumor cells to promote a feed-forward cycle of tumor growth and bone destruction which contributes to the incurability of bone metastases [2]. Hypoxia and high concentrations of TGF-β in the bone microenvironment enhance tumor production of factors that drive the feed-forward cycle of bone metastasis. We asked whether the hypoxia and TGF-β signaling pathways have additive or synergistic effects to promote breast cancer bone metastasis to determine if combined treatment with inhibitors of these pathways could be used to treat bone metastases.

Bone is the largest storehouse of TGF-β in the body. TGF-β has complex effects in cancer and is a growth suppressor early in tumorigenesis; however, many advanced cancers escape from growth inhibition by TGF-β and express prometastatic genes in response [3]. TGF-β signaling pathway is activated when TGF-β binds to the TGF-β type II receptor (TβRII) and promotes dimerization with and activation of the TGF-β type I receptor (TβRI) [3]. TβRI contains a kinase domain which phosphorylates the receptor-associated Smads, Smad2 and Smad3. These factors bind to Smad4 forming a heteromeric Smad complex which translocates to the nucleus and mediates gene transcription by binding to Smad binding elements (SBEs) in the promoters of target genes [4].

TGF-β has an additional role in cancer to promote bone metastasis by regulating many of the tumor-secreted factors that stimulate tumor growth and bone destruction [5] (Table 1), such as PTHrP [6], IL-11, connective tissue growth factor (CTGF), the CXC chemokine receptor 4 (CXCR4), and others [7]–[10]. Previous studies using mouse models have shown that blockade of TGF-β signaling in MDA-MB-231 breast carcinoma cells by stable expression of a dominant-negative TβRII reduced bone metastases and increased survival [6]. Expression of a constitutively active TβRI reversed this effect, resulting in increased bone metastases and decreased survival [6]. Inhibition of TGF-β signaling by knockdown of Smad4 [11], [12], overexpression of the inhibitory Smad7 [13], or treatment with pharmacologic inhibitors, such as SD-208 [14], an ATP-competitive inhibitor of the TβRI kinase or other TGF-β inhibitors [15]–[18] decreased bone metastases in animal models.

**Table 1:** Regulation of bone metastases genes by hypoxia and TGF-β.

Gene Type	Gene	Hypoxia	Both	TGF-β
**Bone Metastatic Genes (Kang ** ***et al.*** ** 2003)**	CTGF	34^*^	6	632^*^
	CXCR4	87^*^	1	55^*^
	OPN	61^*^	4	314^*^
	IL-11	5	0	171^*^
	MMP-1	24^*^	7	216^*^
**Tumor-Secreted Factors**	PTHrP	9	1	193^*^
	IL-6	384^*^	20^*^	2974^*^
	IL-8	200^*^	6	816^*^
	Endothelin-1	590^*^	17^*^	313^*^
	Adrenomedullin	140^*^	3	38^*^
	VEGF	3152^*^	108^*^	1182^*^
	PDGF	128^*^	23	1823^*^
	Stanniocalcin	12^*^	0	3
	Thrombospondin	59^*^	9	314^*^
	Cyr61	17^*^	1	28^*^

Number of hits returned from a Pubmed literature search conducted in February 2009 for the name of each gene with the keywords TGF-β and/or hypoxia. Asterisk (^*^) indicates genes for which regulation by these pathways has been published.

Bone is a hypoxic microenvironment (pO_2_ between 1–7%) [19], which increases growth of metastatic tumor cells adapted for surviving in conditions of low O_2_. Breast cancers and other solid tumors are susceptible to hypoxia because they proliferate and outgrow vascular supplies of oxygen and nutrients [20]. Tumor hypoxia causes radio- and chemotherapeutic resistance, which may contribute to the incurability of bone metastases [21]. Hypoxia activates signaling through hypoxia-inducible factor (HIF)-1α, which is overexpressed in many cancers, including breast. HIF-1α expression correlates with increasing tumor grade, invasion, and metastasis [22]. In conditions of high oxygen, HIF-1α is hydroxylated and targeted for proteasomal degradation by the von Hippel Lindau tumor suppressor. When oxygen is limiting, HIF-1α heterodimerizes with HIF-1β in the nucleus and mediates the transcription of hypoxia-regulated target genes [20], [23].

Many bone metastases genes that are regulated by TGF-β are also regulated by hypoxia (Table 1), including those identified by Kang *et al*. to comprise a bone-metastatic gene signature in breast cancer cells: CTGF, CXCR4, IL-11, and MMP-1 [8], [13], [24]–[26]. These genes code for proteins that regulate different steps of the metastatic cascade: invasion, homing, angiogenesis and osteolysis. Breast cancer cells express there and many other prometastatic genes. Thus, therapeutic targeting of individual proteins is unlikely to cure breast cancer bone metastases. Inhibitors of HIF-1α or TGF-β, which act upstream of multiple target genes, may be more effective and several are under investigation in phase I and II clinical trials for various cancers [14], [15], [27]–[31].

We investigated interactions between the hypoxia and TGF-β signaling pathways *in vitro* by examining bone-metastatic MDA-MB-231 breast cancer cells for changes in TGF-β and hypoxia-stimulated gene expression of 16 candidate genes. Of these, only vascular endothelial growth factor (VEGF) and CXCR4, showed additive responses to TGF-β and hypoxia, suggesting limited crosstalk between TGF-β and hypoxia signaling pathways in breast cancer cells. *In vitro* analyses, however, may not accurately represent *in vivo* function. Therefore, we used a mouse model of bone metastasis to assess global crosstalk between the hypoxia and TGF-β signaling pathways *in vivo*. In this model, the MDA-MB-231 breast cancer cell line reliably forms osteolytic bone lesions in nude mice when inoculated into the left cardiac ventricle. We tested the effects TGF-β and hypoxia on bone metastases in this model by genetic and pharmacologic approaches. In the first approach, we used genetic methods of shRNA knockdown and dominant-negative expression to specifically target the tumor cells. Genetic inhibition of HIF-1α and TGF-β signaling pathways in MDA-MB-231 cells significantly decreased osteolysis and enhanced survival of mice with bone metastases. Combined inhibition of HIF-1α and TGF-β in the tumor cells had no additional effect, suggesting parallel roles for hypoxia and TGF-β signaling in tumor cells. This approach provided proof of principle for the tumor autonomous effects of HIF-1α and TGF-β signaling in bone metastases, but it is not readily translatable to the clinic. In a second pharmacologic approach, we inhibited HIF-1α and TGF-β signaling systemically using small molecule inhibitors to target both the tumor cells and the bone microenvironment. Inhibition of HIF-1α or TGF-β with these inhibitors also decreased osteolysis, reduced tumor burden, and enhanced survival of mice with bone metastases. In contrast to the genetic models, combined pharmacologic inhibition produced an additional decrease in tumor burden compared to either alone. Systemic inhibition of HIF-1α and TGF-β signaling also had independent effects on bone to decrease osteoclast and increase osteoblast activity. Thus, combined systemic inhibition of HIF-1α and TGF-β signaling is more beneficial than either alone due to activity on the tumor cells and the bone microenvironment.

## Results

### Hypoxia induces HIF-1α expression in bone metastatic cancer cell lines in vitro and in vivo at sites of MDA-MB-231 breast cancer bone metastases

**Figure 1:**
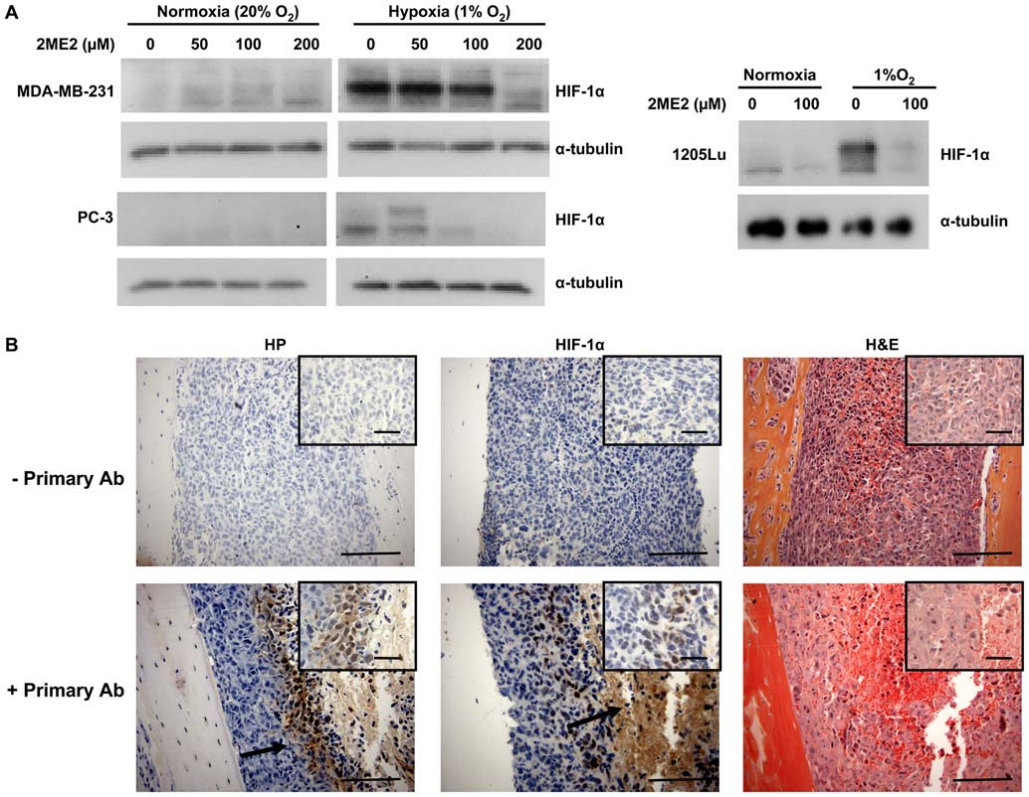
Hypoxia induces HIF-1α in bone-metastatic cancer cells *in vitro* and in bone metastases *in vivo*. (A) MDA-MB-231, PC-3 and 1205Lu cells were treated with 2ME2 and cultured ± 1% O_2_ during 6 h. Protein lysates from the treated cells were analyzed for HIF-1α protein expression by Western blotting. α-tubulin was used as loading control. (B) Representative demineralized bone sections from mice with MDA-MB-231 bone metastases stained for tumor hypoxia with Hypoxyprobe^TM^-1 (HP), with an antibody against HIF-1α, or with hematoxylin and eosin (H&E). Arrows indicate HIF-1α positive nuclei. Images are at 200×magnification with scale bar equal to 100 µm, inset is at 630×magnification with scale bar equal to 50 µm. Staining without primary antibody was used as negative control.

Three bone metastatic cancer cell lines: MDA-MB-231 breast cancer, PC-3 prostate cancer, and 1205Lu melanoma cells, were tested for hypoxic responsiveness by culture in 20% or 1% O_2_ for 6 h. Western blot analysis showed induction of HIF-1α expression under hypoxic conditions, which was blocked by treatment with the HIF-1α inhibitor 2-methoxyestradiol (2ME2) [32]–[34] (Figure 1A). We next determined whether MDA-MB-231 breast cancer bone metastases are hypoxic *in vivo* by Hypoxyprobe™-1 staining [35]–[38]. Mice with MDA-MB-231 bone metastases were injected 2 h prior to euthanasia with pimonidazole, which forms insoluble protein adducts in hypoxic cells. Staining was detected in bone metastases sections from pimonidazole-labeled mice but not in control animals (Figure 1B). Staining for HIF-1α protein was detected in serial bone metastases sections, at sites adjacent to pimonidazole-positive hypoxic regions. The results suggest a role for hypoxia-induced HIF-1α in bone metastases.

### Hypoxia and TGF-β additively increase prometastatic factors VEGF and CXCR4

**Figure 2:**
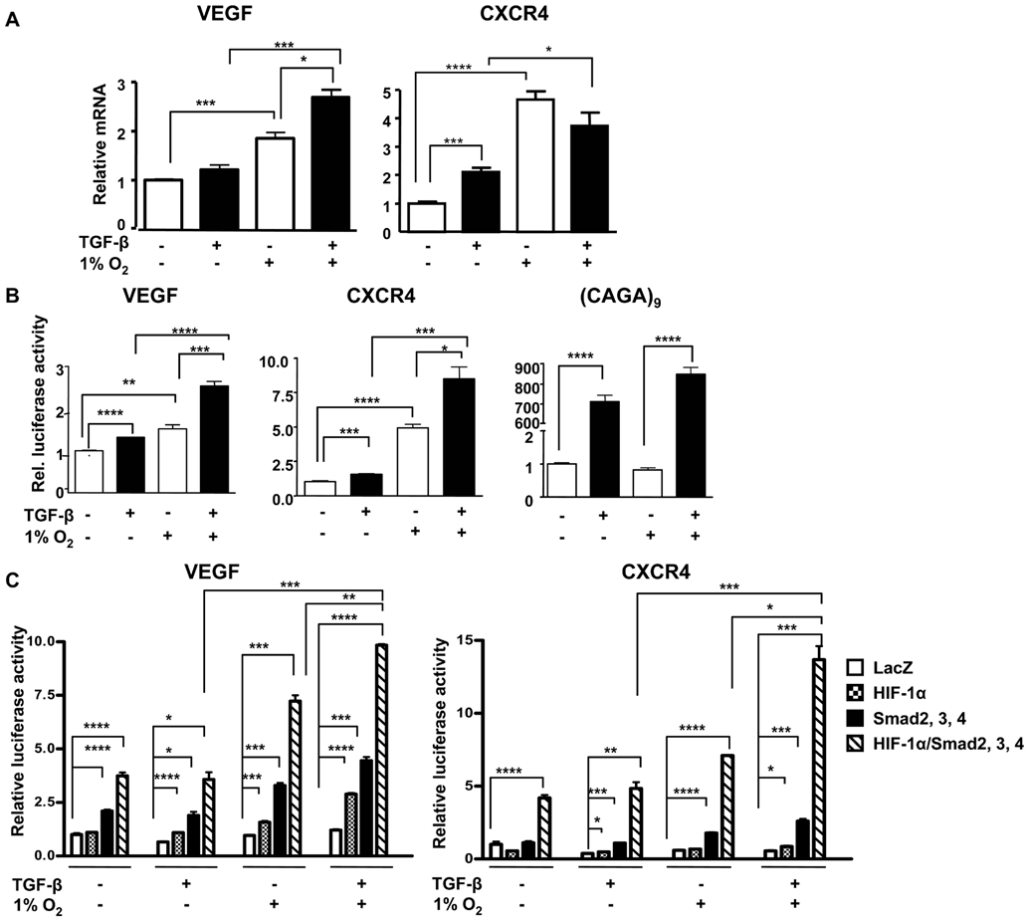
Hypoxia and TGF-β increase VEGF and CXCR4 mRNA expression and promoter activity. (A) MDA-MB-231 cells were cultured ± TGF-β (5 ng/mL) ± 1% O_2_ during 24 h and total RNA was extracted. VEGF and CXCR4 mRNA expression (mean ± SEM) was measured by semi-quantitative RT-PCR (n = 3). * *P<*0.05, *** *P<*0.005, **** *P<*0.001, using an unpaired Student's t test. (B) HepG2 hepatocarcinoma cells co-transfected with a renilla luciferase plasmid (phRL-CMV) and a pGL3 plasmid containing a fragment of the human VEGF (3.3 kb) or CXCR4 (2.6 kb) promoter, or the TGF-β-sensitive (CAGA)_9_ promoter were treated ± TGF-β (5 ng/mL) and ± 1% O_2_ during 24 h before measuring dual-luciferase activity. (C) MDA-MB-231 breast cancer cells were transfected with a pGL3 plasmid containing the human VEGF or CXCR4 promoter and the phRL-CMV plasmid, as well as plasmids to overexpress HIF-1α and/or Smad2, 3 and 4. Cells were treated ± TGF-β (5 ng/mL) ± 1% O_2_ during 24 h before measuring dual-luciferase activity. Results are expressed as the mean ± SEM (n = 3) of the relative luciferase activity. * *P*<0.05, ** *P*<0.01, *** *P*<0.005, **** *P*<0.001, using an unpaired Student's t test.

A literature search for bone metastatic genes combined with the keywords hypoxia or TGF-β, found that many were regulated by the two pathways (Table 1). MDA-MB-231 cells treated ± TGF-β1 (5 ng/mL) and ± 1% O_2_ for 24 h were then surveyed by semi-quantitative RT-PCR for changes in mRNA expression of candidate TGF-β and hypoxia-regulated genes selected from Table 1: VEGF; CXCR4; PTHrP; IL-6, 8, 11; and CTGF. We also measured the expression of components of the two signaling pathways: HIF-1α, prolyl hydroxylase 2 (PHD2), TGF-β1, Smads2, 3, 4 and 7, Ski and SnoN. Many genes were increased by TGF-β or hypoxia alone (data not shown) while only 2 of the 16 surveyed genes, VEGF and CXCR4, were increased by both TGF-β and 1% O_2_ alone (Figure 2A). VEGF mRNA expression was additively increased by TGF-β and 1% O_2_; however, there was no additional increase in CXCR4 mRNA expression with combined treatment. Transcriptional activation of the VEGF and CXCR4 promoters by TGF-β and hypoxia was analyzed by dual-luciferase assay in HepG2 cells transfected with a pGL3-luciferase reporter vector containing either a 3.3 kb human VEGF promoter fragment [39] or a 2.6 kb human CXCR4 promoter fragment [40], [41]. A (CAGA)_9_ promoter-luciferase construct containing nine tandemly-repeated Smad-binding elements (SBEs) was used as positive control for TGF-β activation. Dual-luciferase activity was assayed after a 24 h-treatment ± TGF-β1 and ± 1% O_2._ VEGF and CXCR4 promoter activities were increased by treatment with TGF-β or hypoxia alone (Figure 2B). Combined treatment additively increased VEGF and CXCR4 promoter activation. (CAGA)_9_ promoter activity was increased only by treatment with TGF-β, demonstrating that hypoxia does not directly regulate TGF-β/Smad signaling. These results suggest that crosstalk between the hypoxia and TGF-β signaling pathways regulates VEGF and CXCR4 mRNA expression and promoter activation.

### Overexpression of HIF-1α and Smads increases VEGF and CXCR4 expression

Next we tested whether HIF-1α and Smads mediate promoter activation in response to hypoxia and TGF-β. HIF-1α or Smads2, 3, and 4 were overexpressed in MDA-MB-231 cells cotransfected with either the VEGF or CXCR4 promoter-luciferase plasmids. Dual-luciferase activity was assayed after a 24 h-treatment with TGF-β1 and 1% O_2_. Overexpression of either HIF-1α or Smads increased VEGF and CXCR4 promoter activity in response to TGF-β and hypoxia (Figure 2C). Coexpression of HIF-1α and Smads together resulted in a 10-fold increase in VEGF and CXCR4 promoter activities in the presence of TGF-β and hypoxia, suggesting that VEGF and CXCR4 transcriptional responses to hypoxia and TGF-β are mediated through HIF-1α and Smads, respectively.

### TGF-β and hypoxia interact to regulate VEGF and CXCR4 transcription

**Figure 3:**
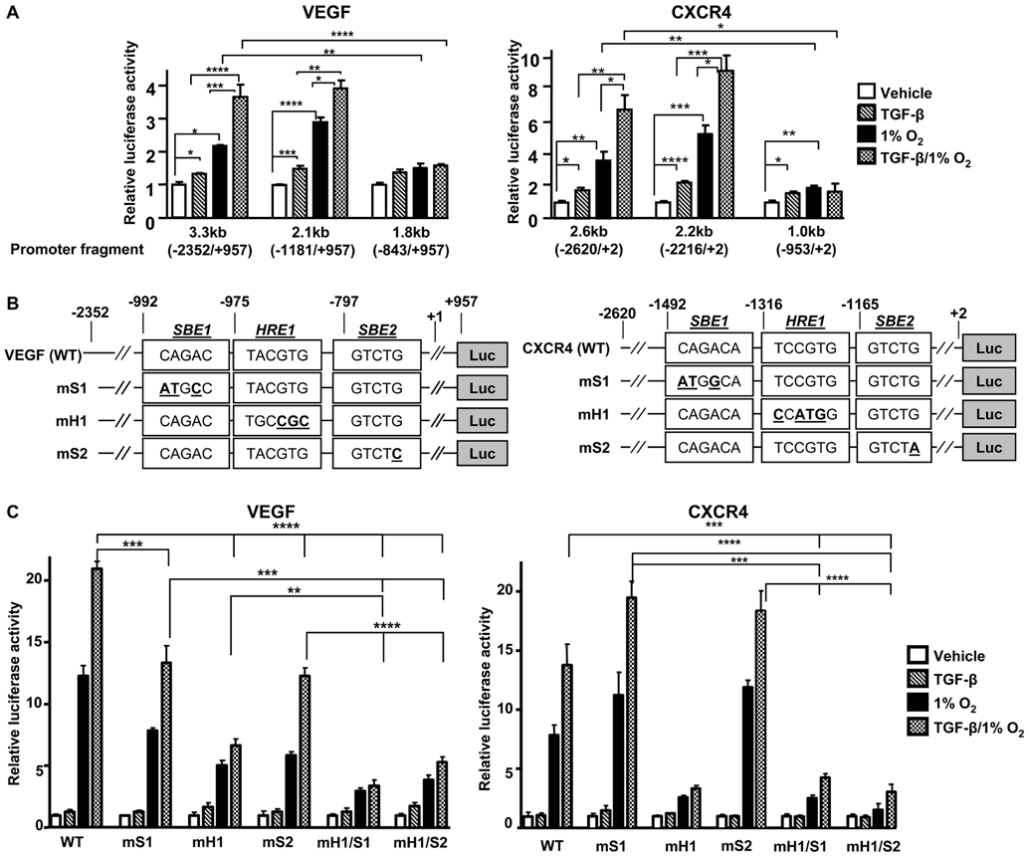
Hypoxia and TGF-β increase VEGF and CXCR4 transcription through proximal promoter response elements. (A) HepG2 cells were transfected with pGL3 plasmids containing full-length for 5′-deleted fragments of the human VEGF or CXCR4 promoter and the phRL-CMV plasmid. Cells were treated ± TGF-β (5 ng/mL) ± 1% O_2_ during 24 h before measuring dual-luciferase activity. Results are expressed as the mean ± SEM (n = 3) of the relative luciferase activity, analyzed using an unpaired Student's t test. (B) Schematic representation of wild-type (WT), HRE-mutant (mH1) and SBE-mutant (mS1 or mS2) VEGF and CXCR4 promoters. One to three nucleotides (underlined, bold letters) within HRE and SBEs were substituted as indicated. (C) HepG2 cells were transfected with pGL3 plasmids containing a wild-type VEGF or CXCR4 promoter or promoters with mutations to the HRE or SBE and the phRL-CMV plasmid. Cells were treated ± TGF-β (5 ng/mL) ± 1% O_2_ during 24 h before measuring dual-luciferase activity. Results are expressed as the mean ± SEM (n = 3) of the relative luciferase activity, analyzed using an unpaired Student's t test. * *P*<0.05, ** *P*<0.01, *** *P*<0.005, **** *P*<0.001.

To identify promoter regions targeted by TGF-β and hypoxia signaling, constructs with 5′-deletion of the promoter, ranging in size from 3.3 kb to 1.8 kb for VEGF and 2.6 kb to 1.0 kb for CXCR4, were tested for TGF-β and hypoxia responsiveness by dual-luciferase assay. VEGF promoter response to TGF-β and hypoxia was blocked by deletion of bases −1181 to −843, while deletion of bases −2216 to −953 blocked CXCR4 responsiveness (Figure 3A). These results suggest that transcriptional activation by TGF-β and hypoxia is localized within 300 base pairs in the VEGF promoter and in a 1.2 kb region for CXCR4.

Promoter sequences were scanned for putative hypoxia response elements (HREs) and SBEs using the consensus sequences 5′-RCGTG-3′ and 5′-CAGAC-3′, respectively. HREs and SBEs found in close proximity within the promoter regions identified above were mutated by site-directed mutagenesis (Figure 3B). TGF-β and hypoxia responsiveness were assayed using dual-luciferase. In the VEGF promoter, mutation of HRE (−975/−970) and SBEs (−992/−988) and (−797/−793) decreased the response to TGF-β and 1% O_2_ (Figure 3C). The combined mutation of the HRE and one of the SBE nearly abolished promoter activity. Mutation of HRE (−1316/−1310) in the CXCR4 promoter blocked additive responses to TGF-β and hypoxia, while mutation of putative SBEs had little or no effect (Figure 3C). These data suggest that both TGF-β and hypoxia regulate VEGF promoter activity, while only hypoxia regulates CXCR4 promoter activity. The promoter may contain additional non-identified SBEs that mediate its response to TGF-β.

### HIF-1α knockdown in MDA-MB-231 breast cancer cells decreases osteolytic bone metastases and improves survival in a mouse model

**Figure 4:**
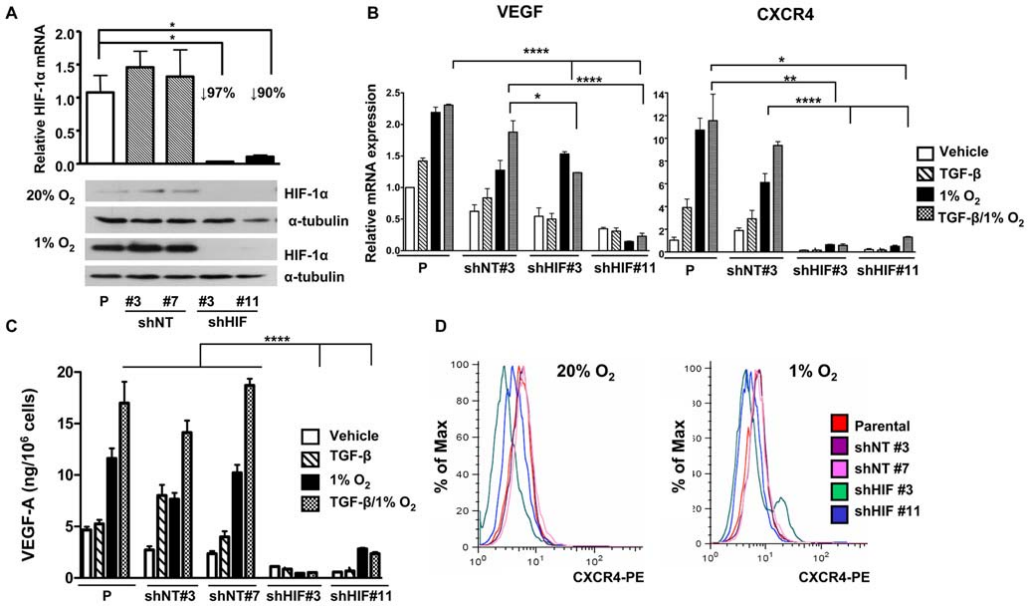
Knockdown of HIF-1α inhibits VEGF and CXCR4 mRNA and protein expression *in vitro*. (A) MDA-MB-231 parental cells (P) or cells transfected with a pLKO.1 vector expressing a non-target shRNA (shNT#3 and #7) or an shRNA against HIF-1α (shHIF#3 and #11) were cultured ± 1% O_2_ during 6 h. Total RNA was extracted and mean ± SEM expression of HIF-1α was measured using semi-quantitative RT-PCR (n = 3). Proteins were extracted from treated cells and HIF-1α level was assayed by Western-blotting, α-tubulin was used as loading control. * *P*<0.05, using an unpaired Student's t test. (B) Parental (P) cells and shNT and shHIF clones were treated ± TGF-β (5 ng/mL) ± 1% O_2_ for 24 h. Total RNA was extracted and mean ± SEM VEGF and CXCR4 expression was measured using semi-quantitative RT-PCR (n = 3). * *P<*0.05, ** *P<*0.01, **** *P<*0.001, using an unpaired Student's t test. (C) MDA-MB-231 parental (P) cells and clones were treated ± TGF-β (5 ng/mL) ± 1% O_2_ and conditioned media were collected 24 h later. VEGF-A levels were measured by ELISA assay. Results are expressed as mean ± SEM nanograms VEGF-A per 10^6^ cells. **** *P<*0.001, using an unpaired Student's t test. (D) MDA-MB-231 parental (P) cells and clones were treated ± 1% O_2_ for 24 h and then analyzed by flow cytometry for CXCR4 protein expression. Results are reported as the percentage of maximal CXCR4 expression.

We further examined hypoxic responses of MDA-MB-231 cells *in vitro* by stable knockdown of HIF-1α. The cells were transfected with a plasmid expressing an shRNA targeting HIF-1α. Single cell clones were isolated and stability of the knockdown was tested after cultivating the cells for 60 days in absence of antibiotic. Two clones (shHIF#3 and #11) with >90% decrease of HIF-1α mRNA and undetectable levels of HIF-1α protein were further analyzed (Figure 4A). Two control shRNA clones (shNT#3 and #7) had HIF-1α mRNA expression similar to parental cells and were used as controls. The clones were also analyzed by semi-quantitative RT-PCR for changes in mRNA expression following TGF-β1 and 1% O_2_ treatment for 24 h. TGF-β- and hypoxia-induced VEGF and CXCR4 mRNA expression was significantly decreased in the shHIF clones compared to parental and shNT cells (Figure 4B). Secreted VEGF-A protein, measured by ELISA (Figure 4C), and CXCR4 protein, measured by flow cytometry (Figure 4D), were also decreased. The results demonstrate that knockdown of HIF-1α blocks expression of prometastatic factors VEGF and CXCR4 in MDA-MB-231 cells. An MTT assay showed no difference in proliferation for any of the shHIF or shNT clones compared to parental MDA-MB-231 cells in normoxic conditions. Proliferation of each clone was decreased by culture in hypoxic compared to normoxic conditions; however, there was no difference among the clones (data not shown).

**Figure 5:**
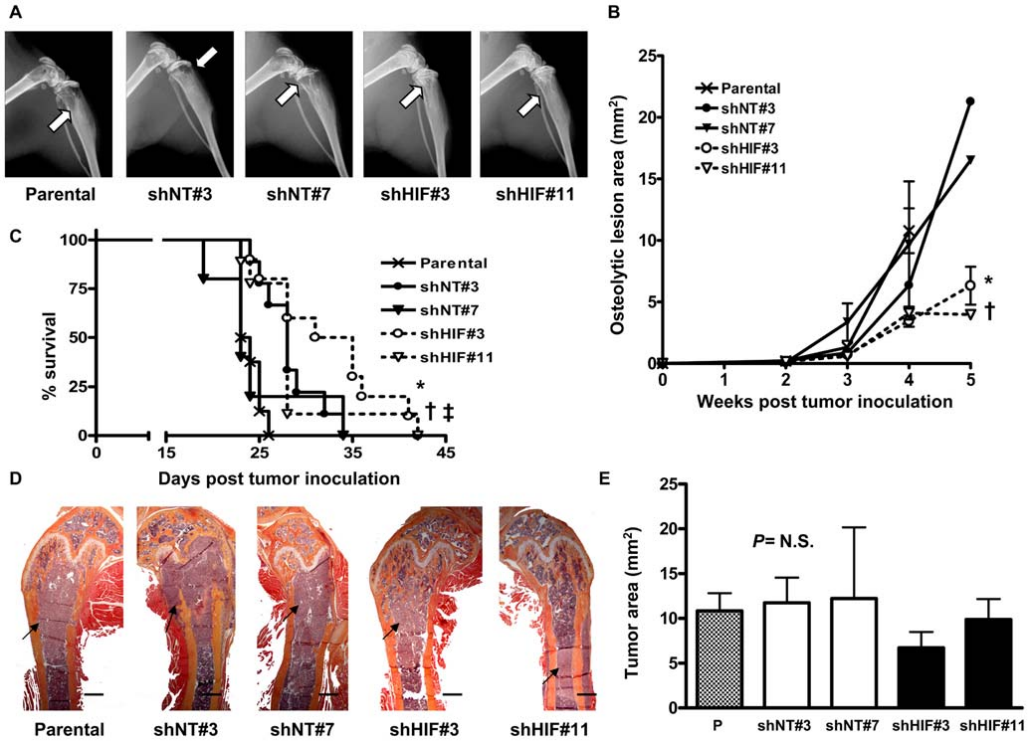
Knockdown of HIF-1α decreases osteolytic lesions and improves survival of mice *in vivo*. (A) Representative x-ray images from hindlimbs of mice 4 weeks post inoculation with MDA-MB-231 parental, shNT and shHIF cells. Arrows indicate osteolytic lesions. (B) Osteolytic lesion area measured on radiographs of hindlimbs and forelimbs of mice with bone metastases. Results are expressed as the mean area ± SEM per mouse (n = 5–10 per group). † *P<*0.01 and * *P<*0.001 compared to parental or shNT clones using a two-way ANOVA with a Bonferroni post-test at 4 weeks. (C) Kaplan-Meyer analysis of mouse survival. * *P<*0.05 shHIF#3 compared to Parental or shNT clones, † *P<*0.005 and ‡ *P = *0.089 shHIF#11 respectively compared to Parental and shNT#7 using a Logrank test. (D) Representative histology of femurs with tumor indicated by arrows. Scale bar equal to 500 µm. (E) Tumor burden in hindlimbs was measured by quantitative histomorphometry. Results are expressed as the mean ± SEM area per bone. A one-way ANOVA with a Newman-Keuls multiple comparison test showed no significant differences (*N.S.*) between groups.

We tested the effect of HIF-1α knockdown *in vivo* using a mouse model of bone metastases. Female nude mice (4 weeks old) were inoculated in the left cardiac ventricle with parental MDA-MB-231 cells or one of the four different clones: shNT#3 and #7 controls, or shHIF#3 and #11 HIF-1α knockdown clones (n = 12 per group). Osteolytic lesion area on x-ray was decreased in mice with shHIF knockdown bone metastases compared to those with controls of parental or shNT bone metastases (*P<*0.001 for shHIF#3 and *P<*0.01 for shHIF#11 compared to parental or shNT clones) (Figure 5A–B). HIF-1α knockdown in bone metastatic cells also significantly improved survival of mice compared to control animals (Figure 5C). A secondary endpoint for this survival experiment was tumor burden, which was analyzed by quantitative histomorphometry. We observed no difference in tumor burden at time of death for mice with shHIF bone metastases compared to those with parental or shNT bone metastases (Figure 5D–E). We were unable to statistically compare tumor burden in mice from these groups at the same time point, as mice with shHIF bone metastases lived longer than the parental or shNT controls.

**Figure 6:**
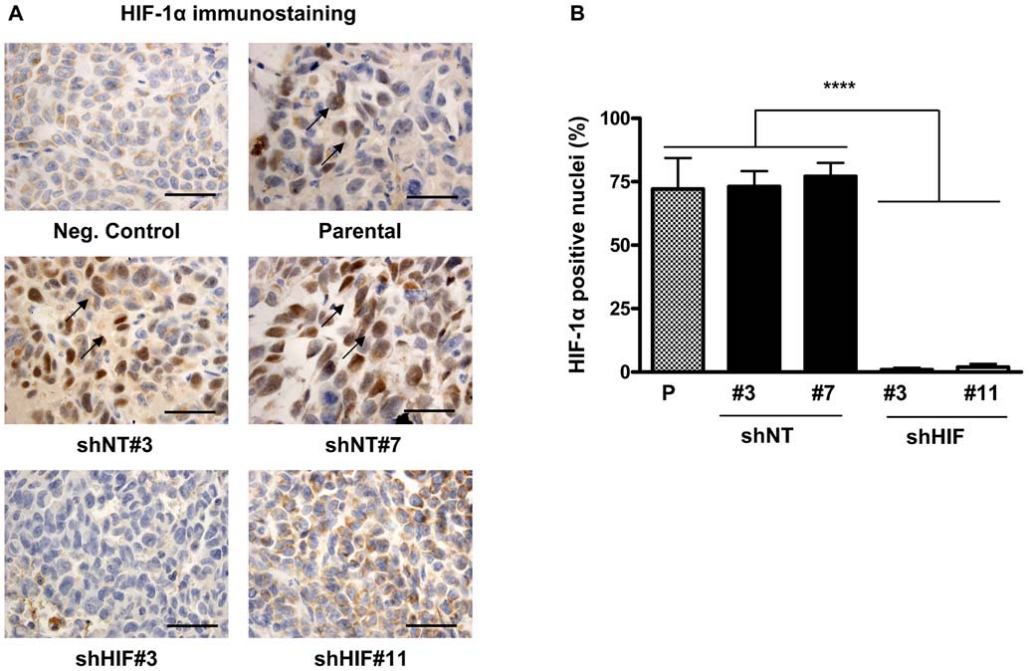
Knockdown of HIF-1α in MDA-MB-231 cells is stable *in vivo*. (A) Representative HIF-1α staining in demineralized bone sections from mice inoculated with MDA-MB-231 parental, shNT or shHIF cells. Arrows indicate HIF-1α positive nuclei. Scale bar equals 50 µm. Staining without primary antibody was used as negative control (Neg. Control). (B) The percentage of nuclei positive for HIF-1α was calculated in three non-overlapping fields at 400×magnification for each mouse (n = 3 per group). Results are the mean ± SEM number of HIF-1α positive nuclei per field.

**Figure 7:**
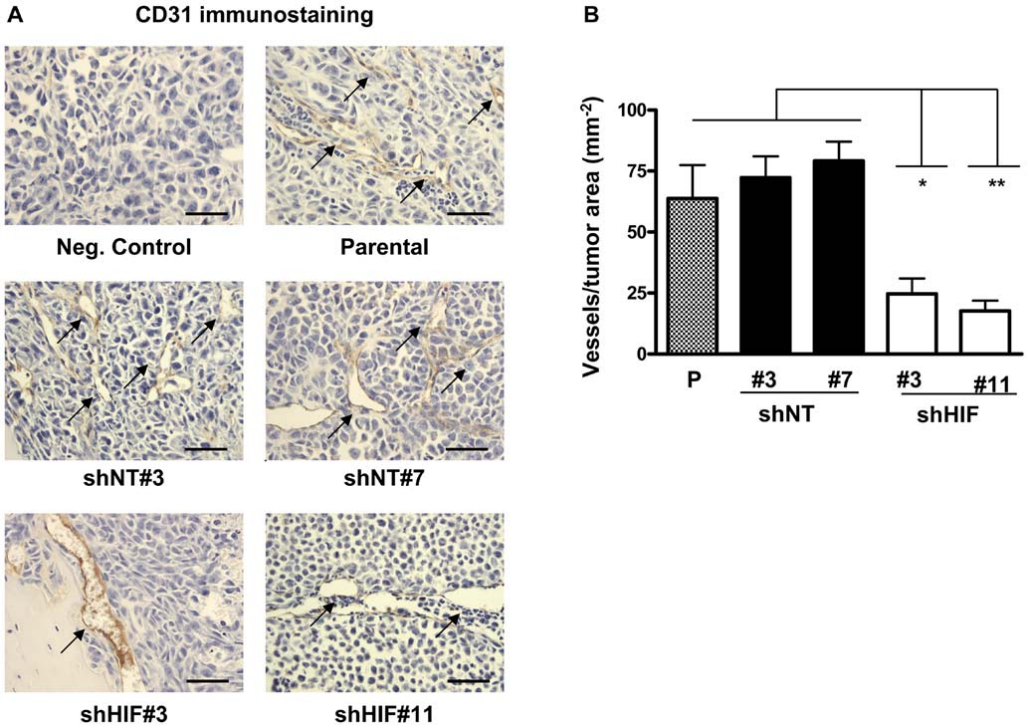
HIF-1α knockdown decreases tumor angiogenesis at sites of bone metastases in mice. (A) Representative sections from mice with bone metastases stained for the endothelial cell marker CD31. Arrows indicate CD31+ vessels. Scale bar equals 50 µm (B) The number of vessels in bone metastases was counted in three non-overlapping fields at 200×magnification per mouse (n = 3 per group). Results are expressed as the mean ± SEM number of vessel per tumor area. * *P<*0.05, ** *P<*0.01, **** *P<*0.001, using a one-way ANOVA with a Newman-Keuls multiple comparison test.

Staining for HIF-1α in bone metastases tumor sections was decreased in shHIF bone metastases compared to parental and shNT controls, confirming the stability of HIF-1α knockdown throughout the *in vivo* experiment (Figures 6A–B). Since HIF-1α knockdown *in vitro* resulted in decreased mRNA expression of the angiogenic factor VEGF, we analyzed tumor angiogenesis *in vivo* by staining for the endothelial cell marker CD31. The number of vessels/tumor area was significantly decreased in shHIF bone metastases compared to parental and shNT bone metastases (Figure 7A–B). These data suggest that knockdown of HIF-1α in tumor cells decreases bone metastases by suppressing tumor secretion of proangiogenic factors and blocking vessel formation.

### HIF-1α knockdown or TGF-β blockade in tumor cells reduces bone metastases and improves survival in vivo

**Figure 8:**
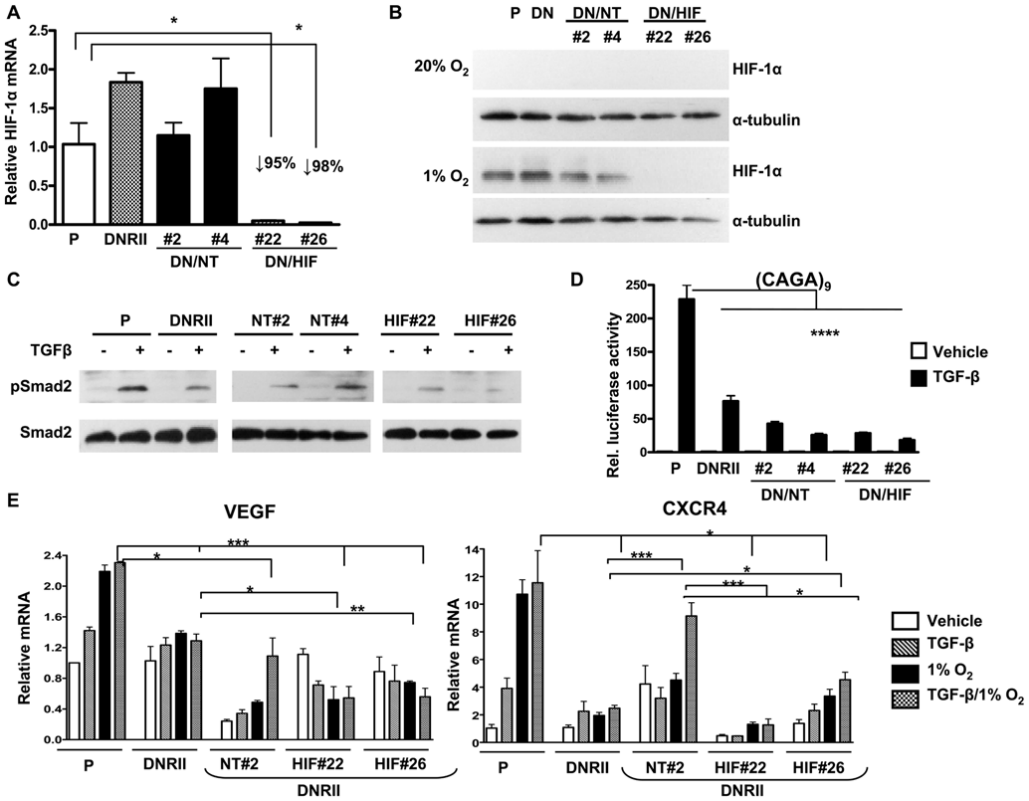
HIF-1α knockdown and TGF-β blockade decreases VEGF and CXCR4 mRNA expression *in vitro*. (A) MDA-MB-231 dominant-negative TβRII expressing cells (DNRII) were transfected with a pLKO.1 vector expressing a non-target shRNA (DNRII/shNT#2 and #4) or an shRNA against HIF-1α (DNRII/shHIF#22 and #26) were cultured ± 1% O_2_ during 6 h. Total RNA was extracted and mean ± SEM expression of HIF-1α was measured using semi-quantitative RT-PCR (n = 3). * *P*<0.05 compared to parental (P), using an unpaired Student's t test. (B) Proteins were extracted from treated cells and HIF-1α level was assayed by Western-blotting. α-tubulin was used as loading control. (C) MDA-MB-231 parental cells (P) and DNRII clones were treated ± TGF-β (5 ng/mL) for 2 h. Proteins were extracted and analyzed for phosphorylated Smad2 (pSmad2) and total Smad2 by Western-blotting. (D) MDA-MB-231 parental cells (P) and DNRII clones transfected with pGL3-(CAGA)_9_ and phRL-CMV plasmids were treated ± TGF-β (5 ng/mL) during 24 h before measuring dual-luciferase activity. Results are expressed as the mean ± SEM of the relative luciferase activity (n = 3). **** *P*<0.001, using an unpaired Student's t test. (E) Total RNA was extracted from MDA-MB-231 parental (P) and DNRII clones treated ± TGF-β (5 ng/mL) and ± 1% O_2_ during 24 h. Mean ± SEM VEGF and CXCR4 expression was measured using semi-quantitative RT-PCR (n = 3). * *P*<0.05, ** *P*<0.01, *** *P*<0.005, using an unpaired Student's t test.

**Figure 9:**
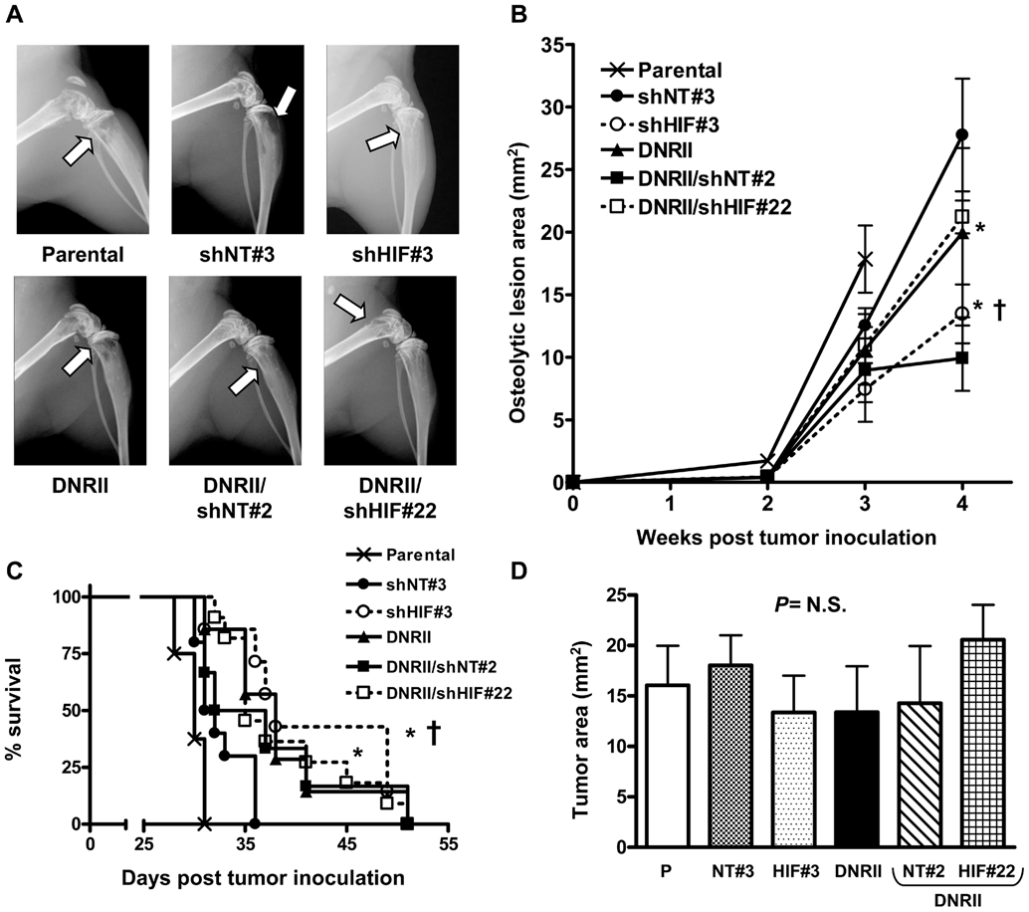
Combined HIF-1α knockdown and TGF-β blockade provides no further benefit in mice with bone metastases. (A) Representative x-ray images from hindlimbs of mice 4 weeks post inoculation with MDA-MB-231 parental, shNT#3, shHIF#3, DNRII, DNRII/shNT#2 and DNRII/shHIF#22 cells. Arrows indicate osteolytic lesions. (B) Osteolytic lesion area measured on radiographs of hindlimbs and forelimbs of mice with bone metastases. Results are expressed as the mean area ± SEM per mouse (n = 6–11 per group). * *P<*0.001 compared to parental and † *P<*0.05 compared to shNT#3 using a two-way ANOVA with a Bonferroni post-test at 3 weeks. (C) Kaplan-Meyer analysis of mouse survival. * *P<*0.001 shHIF#3 and DNRII compared to Parental and † *P<*0.005 shHIF#3 compared to shNT#3 using a Logrank test. (D) Tumor burden in hindlimbs was measured by quantitative histomorphometry. Results are expressed as the mean ± SEM area per bone. A one-way ANOVA with a Newman-Keuls multiple comparison test showed no significant differences (*N.S.*) between groups.

To evaluate the effect of single and combined inhibition of HIF-1α and TGF-β specifically in tumor cells *in vivo*, we generated a series of MDA-MB-231 cell lines which stably expressed either HIF-1α shRNA alone or in combination with a dominant-negative TGF-β type II receptor (DNRII). Knockdown of HIF-1α mRNA and protein was confirmed by semi-quantitative RT-PCR and Western blot (Figure 8A–B). Blockade of TGF-β signaling in the DNRII expressing clones was confirmed by decreased phosho-Smad2 on Western blot (Figure 8C) and decreased (CAGA)_9_ promoter activation in response to TGF-β (Figure 8D). TGF-β and 1% O_2_-stimulated VEGF and CXCR4 mRNAs were decreased in the DNRII and DNRII/shHIF clones compared to parental control (Figure 8E). *In vivo*, mice inoculated with the DNRII/shHIF-1α clones had decreased osteolytic lesion area and improved survival compared to mice with bone metastases caused by parental or DNRII cell lines (data not shown). DNRII/shHIF#22 and DNRII/shNT#2 control clones were selected for a subsequent experiment in which we directly compared the effect of HIF-1α knockdown alone or combined with TGF-β blockade *in vivo*. Female nude mice (n = 12 per group) were inoculated in the left cardiac ventricle with one of the six MDA-MB-231 cell lines: parental, shNT#3, shHIF#3, DNRII, and DNRII/shNT#2 or DNRII/shHIF#22. Mice were followed by radiography for the development of osteolytic lesions. Lesion area on x-ray was decreased by knockdown of HIF-1α (shHIF#3) or blockade of TGF-β (DNRII or DNRII/shNT#2) (*P<*0.001 shHIF#3 and DNRII compared to parental and *P<*0.05 shHIF#3 compared to shNT#3) (Figure 9A–B). Combined inhibition of TGF-β and HIF-1α (DNRII/shHIF#22) had no additional effect on osteolysis. Survival of mice with MDA-MB-231 bone metastases was improved with HIF-1α knockdown or TGF-β blockade (Figure 9C). Combined inhibition of these pathways yielded no further improvement in survival. Quantitative histomorphometry for tumor burden was analyzed as a secondary endpoint for this experiment. There was no difference in tumor burden at time of death in the mice with shHIF, DNRII, or shHIF/DNRII bone metastases compared to the control groups (Figure 9D), which was not unexpected because the survival of these mice was increased.

To determine if the observed effects were bone specific, we analyzed tumor growth following inoculation of these clones into the mammary fat pad. Tumor take and rate of growth were similar for the parental, DNRII, and DNRII/shNT#2 clones, but decreased in the shHIF#3 and DNRII/shHIF#22 clones (data not shown). The data suggest that HIF-1α knockdown may decrease bone metastases by inhibiting tumor cell proliferation rather than increasing apoptosis, as TUNEL staining of bone metastases tumor sections demonstrated no difference in tumor cell apoptosis in shHIF compared to parental or shNT bone metastases (data not shown).

### Pharmacologic inhibition of HIF-1α with 2ME2 decreases osteolytic lesion area and tumor burden in a preventive model of bone metastasis

The preceding studies provide proof of principle that the HIF-1α and TGF-β signaling pathways in breast cancer cells promote skeletal metastases. Molecular blockade of either pathway prevents tumor growth in bone although the effects were not additive. To determine whether systemic inhibition of these pathways in tumor and host cells provided similar benefit, we used a pharmacologic approach with the HIF-1α inhibitor, 2-methoxyestradiol (2ME2) [32]–[34]. We showed that 2ME2 decreases HIF-1α protein expression in MDA-MB-231 breast cancer cells *in vitro* (Figure 1A). 2ME2 was also previously shown to decrease osteolysis in a 4T1 breast cancer metastasis model [42]. Here, we tested a nanocrystalline dispersion formulation with improved bioavailability [43] in a prevention model for breast cancer bone metastases. Drug treatment (150 mg/kg i.p.) was initiated two days prior to the inoculation of tumor cells and continued daily during the experiment. Female nude mice (n = 15 per group) were inoculated into the left cardiac ventricle with MDA-MB-231 cells and the development of osteolytic lesions was followed by radiography. Animals were euthanized at the same time point in order to compare tumor burden between the vehicle and 2ME2 treated groups.

**Figure 10:**
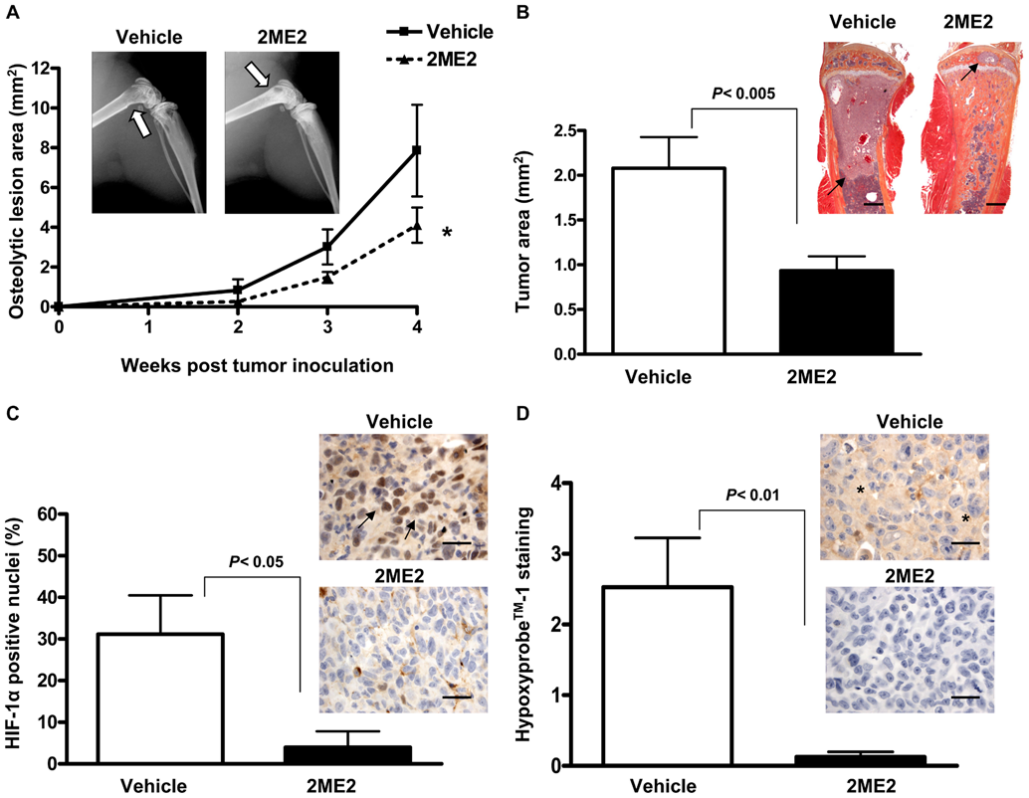
Preventive treatment with the HIF-1α inhibitor 2ME2 inhibits bone metastases in mice. (A) Representative 4-week x-rays from mice inoculated with MDA-MB-231 cells and treated ± 2ME2 (150 mg/kg), arrows indicate osteolytic lesions. Osteolytic lesion area was measured on radiographs of hindlimbs and forelimbs of mice with bone metastases. Results are expressed as the mean ± SEM area per mouse (n = 10–12 per group). * *P<*0.01 using a two-way ANOVA with a Bonferroni post-test at 4 weeks. (B) Representative histology of tibias with tumor indicated by arrows. Scale bar equal to 500 µm. Tumor burden in hindlimbs and humeri was measured by quantitative histomorphometry. Results are expressed as the mean ± SEM area per bone, analysis by unpaired Student's t test. (C) Representative HIF-1α staining in demineralized bone metastases sections from mice treated ± 2ME2. Arrows indicate HIF-1α positive nuclei. Scale bar equals 25 µm. The percentage of nuclei positive for HIF-1α was calculated in three non-overlapping fields at 400×magnification for each mouse (n = 6 per group). Results are the mean ± SEM number of HIF-1α positive nuclei per field using an unpaired Student's t test. (D) Hypoxyprobe^TM^-1 staining for tumor hypoxia in bone metastases sections. Hypoxic regions indicated by asterisks. Staining was graded on a 1–4+ scale in three non-overlapping fields at 400×magnification per mouse (n = 3 per group). Results are the mean ± SEM staining per field using an unpaired Student's t test.

Preventive treatment with 2ME2 significantly reduced osteolysis area (*P<*0.01) (Figure 10A) with a consistent decrease in tumor burden by histomorphometric analyses (*P<*0.005) (Figure 10B). Staining for nuclear HIF-1α in the tumor cells was significantly decreased in mice treated with 2ME2 compared to vehicle-treated mice, confirming that the 2ME2 treatment was effective *in vivo* (Figure 10C). Tumor hypoxia was also decreased in 2ME2-treated mice as demonstrated by Hypoxyprobe^TM^-1 staining (Figure 10D). The data indicate that 2ME2 effectively inhibits HIF-1α expression within the tumor microenvironment.

### 2ME2 increases bone mass by increasing osteoblasts and inhibiting osteoclasts in bone unaffected by tumor

**Figure 11:**
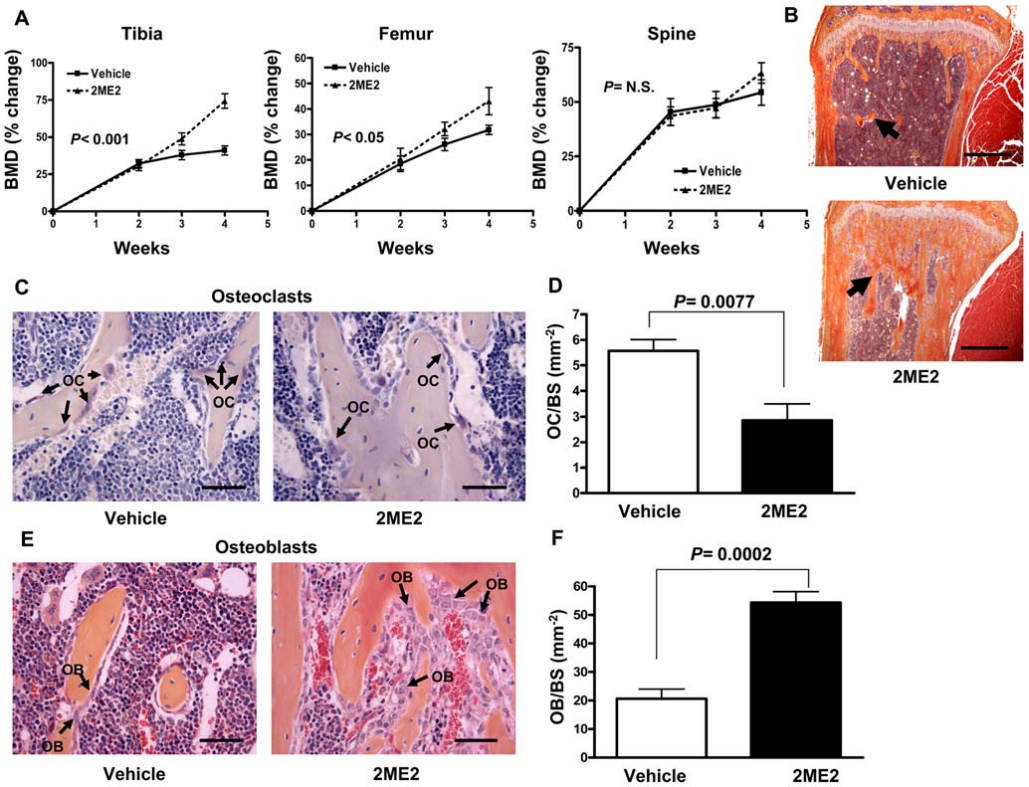
2ME2 increases bone density, inhibits osteoclasts and increases osteoblasts in normal bone unaffected by tumor. (A) Bone mineral density of the femur, tibia, and spine measured by DXA in mice treated ± 2ME2 (150 mg/kg). Results are expressed as the mean ± SEM % change in BMD (n = 10 per group). Statistical analysis by two-way ANOVA with a Bonferroni post-test at week 4. (B) Representative histology of tibias from mice ± 2ME2 (150 mg/kg) for 4 weeks. Trabecular bone is indicated by arrows. Scale bar equal to 500 µm. (C) Representative TRAP staining of bone histology of the proximal tibias from the mice. TRAP+ osteoclasts (OC) are indicated by arrows. Scale bar is equal to 50 µm. (D) Osteoclast number was measured in the distal femur and proximal tibia at 200×magnification on TRAP stained slides. Results are expressed as the number of osteoclasts (OC) per mm^2^ bone surface (BS). (E) Representative H&E stained bone histology of the proximal tibias from mice treated ± 2ME2 (150 mg/kg). Osteoblasts (OB) indicated by arrows. Scale bar is equal to 50 µm. (F) Osteoblast number was measured below the primary spongiosa in the distal femur and proximal tibia at 200×magnification on H&E stained slides. Results are expressed as the number of osteoblasts (OB) per mm^2^ bone surface (BS).

To determine the effects of 2ME2 in normal bone, mice were treated with 2ME2 (150 mg/kg i.p) daily for 4 weeks. Bone mineral density (BMD) was measured weekly by DXA throughout the experiment. Mice treated with 2ME2 had significantly increased BMD at the tibia (*P<*0.001) and femur (*P<*0.05), but not at the spine (Figure 11A). Trabecular bone was increased at the distal femur and proximal tibia by 2ME2 treatment, as demonstrated by histology of bones from the mice (Figure 11B). To determine if alterations in bone resorption or bone formation were responsible for the increase bone mass, we counted osteoclast and osteoblast on histologic sections of bone using histomorphometric analysis. Osteoclast number per bone surface was decreased (*P = *0.0077, Figure 11C–D), while osteoblast number was increased (*P = *0.0002, Figure 11E–F) in the 2ME2-treated mice compared to vehicle-treated controls. The data suggest that 2ME2 may inhibit bone metastases through direct effects on bone by inhibiting osteoclast and increasing osteoblast activity, in addition to its effects on tumor cells.

### Pharmacologic inhibition of TβRI reduces the development and progression of breast cancer metastases to bone and improves survival

**Figure 12:**
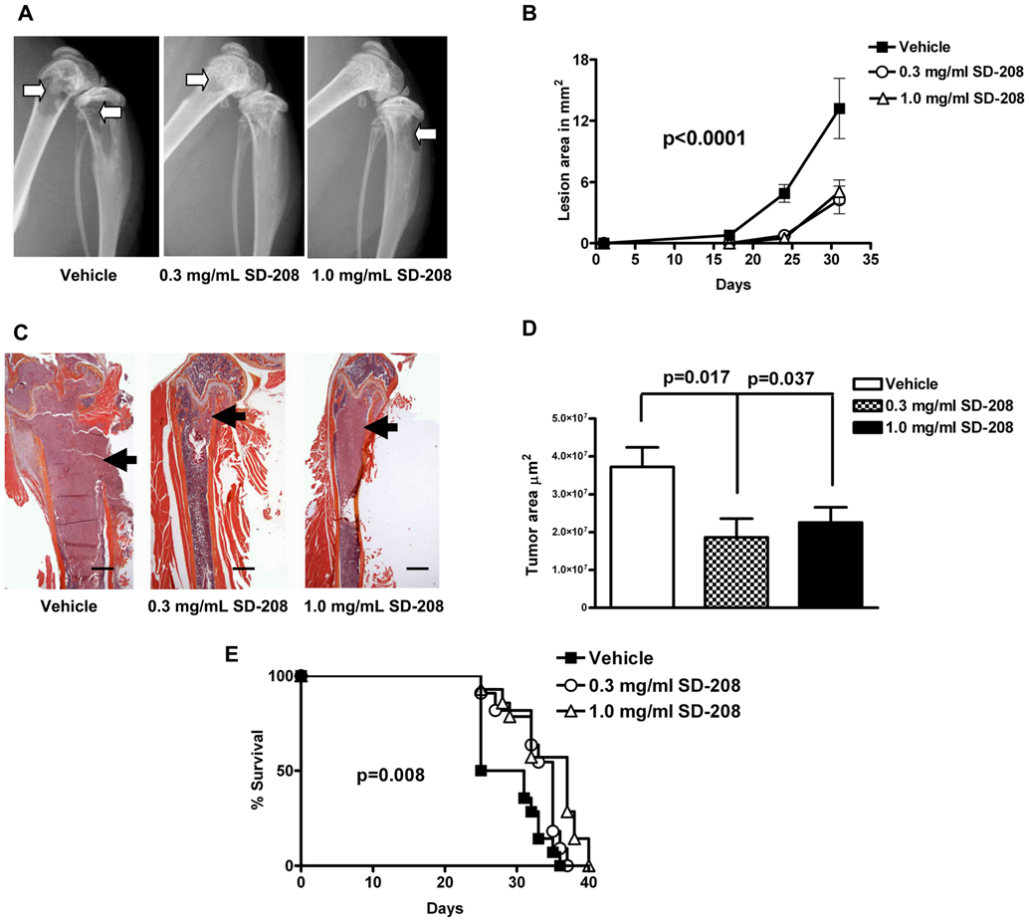
Preventive treatment with SD-208 reduces osteolytic bone lesions and increases survival. (A) Representative x-ray images from hindlimbs of mice inoculated with MDA-MB-231 cells and treated ± SD-208 (0.3 mg/mL or 1.0 mg/mL), arrows indicate osteolytic bone lesions. (B) Osteolytic lesion area measured on radiographs of hindlimbs and forelimbs from mice with bone metastases. Results are expressed as the mean area ± SEM per mouse. **** *P<*0.0001 0.3 mg/mL and 1.0 mg/mL SD-208 compared to vehicle, using a two-way ANOVA with a Bonferroni post-test at 4 weeks. (C) Representative histology of femurs with tumor indicated by arrows. Scale bar equal to 500 µm. (D) Tumor burden in hindlimbs and humeri was measured by quantitative histomorphometry. Results are expressed as the mean ± SEM area per bone. Statistical analysis by one-way ANOVA with a Newman-Keuls multiple comparison test. (E) Kaplan-Meyer analysis of mouse survival analyzed using a Logrank test.

To compare single systemic inhibition of HIF-1α with inhibition of TGF-β signaling, we tested the efficacy of SD-208, a small molecule inhibitor of TβRI kinase, in a prevention model of bone metastasis. Mice were treated with either 0.3 or 1.0 mg/mL SD-208 in the drinking water, beginning two days prior to tumor inoculation and continuing for the duration of the study. Both doses of SD-208 significantly reduced the osteolytic lesions detectable on radiography (Figure 12A–B). Histomorphometric analysis of total tumor area showed a similar reduction in tumor burden (Figure 12C–D). Furthermore, SD-208 treatment resulted in a dose-dependent increase in median survival compared to control animals (25, 35 and 37 days for vehicle, 0.3 and 1.0 mg/mL SD-208, respectively, p = 0.008) (Figure 12E).

### Combined inhibition of HIF-1α and TGF-β with small molecule inhibitors additively decreases bone metastases in mice

**Figure 13:**
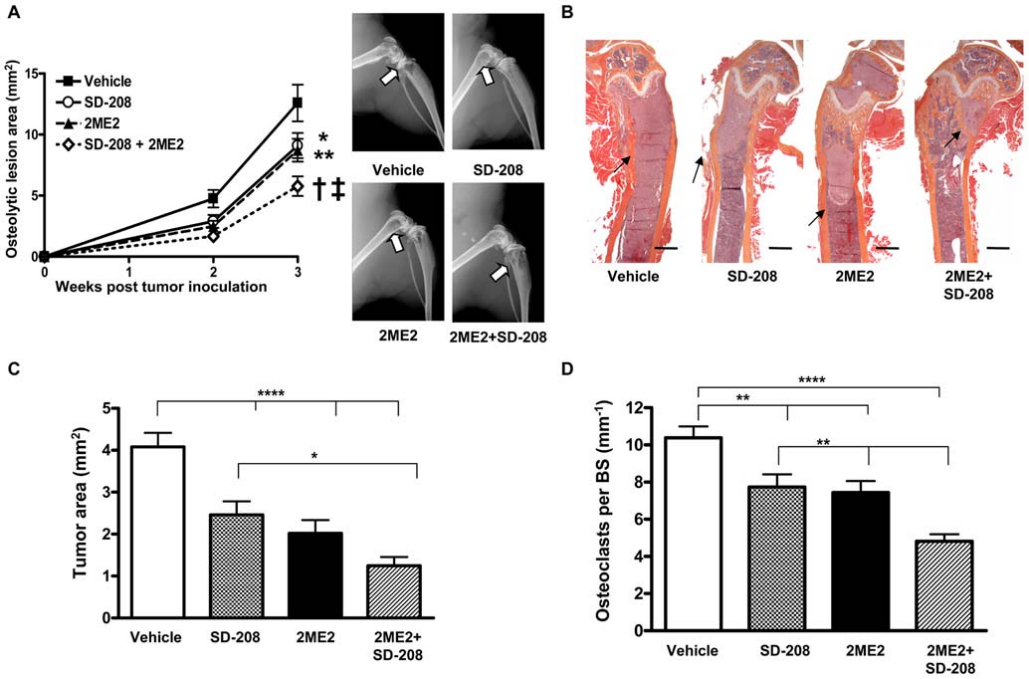
Combined therapeutic treatment with 2ME2 and SD-208 additively decreases bone metastases in mice. (A) Representative x-ray images from hindlimbs of mice inoculated with MDA-MB-231 cells and therapeutically treated ± 2ME2 (150 mg/kg) and ± SD-208 (60 mg/kg), arrows indicate osteolytic lesions and osteolytic lesion area measured on radiographs of hindlimbs and forelimbs from mice with bone metastases. Results are expressed as the mean area ± SEM per mouse (n = 12–14 per group). * *P<*0.01 SD-208 and ** *P<*0.001 2ME2 compared to vehicle, † *P<*0.01 SD-208 and ‡ *P<*0.05 2ME2 compared to SD-208 + 2ME2, using a two-way ANOVA with a Bonferroni post-test at 3 weeks. (B) Representative histology of femurs with tumor indicated by arrows. Scale bar equal to 500 µm. (C) Tumor burden in hindlimbs and humeri was measured by quantitative histomorphometry. Results are expressed as the mean ± SEM area per bone. * *P<*0.05, **** *P<*0.001, using a one-way ANOVA with a Newman-Keuls multiple comparison test. (D) Osteoclast number per mm bone surface (BS) was counted in TRAP stained bone metastases sections. Results are expressed as mean ± SEM OC number per millimeter BS per bone. ** *P<*0.01, **** *P<*0.001, using a one-way ANOVA with a Newman-Keuls multiple comparison test.

Next, we tested whether combined systemic inhibition of HIF-1α with 2ME2 and TGF-β with a TβRI kinase inhibitor SD-208 [14], [31] provided additional therapeutic benefit in a therapeutic model of breast cancer bone metastasis. Mice were inoculated with MDA-MB-231 cells and were followed by x-ray for the development of bone metastases. Treatment was initiated when osteolytic lesions were observed on x-ray at 12 days post tumor inoculation and continued daily during the experiment. Mice (n = 15/group) were randomized to one of four treatment groups: vehicle, 2ME2 alone (150 mg/kg i.p.), SD-208 alone (60 mg/kg by oral gavage), or 2ME2 and SD-208 combined. To control for the effects of the different methods of drug administration, all mice received daily i.p. injection with either 2ME2 or PBS vehicle and oral gavage with either SD-208 or 1% methylcellulose vehicle. Treatment with either 2ME2 or SD-208 alone significantly decreased x-ray lesion area (*P<*0.01 SD-208 and *P<*0.001 2ME2 compared to vehicle) (Figure 13A), which was further decreased with combined treatment (*P<*0.01 and *P<*0.05 SD-208 + 2ME2 compared to SD-208 and 2ME2, respectively). Mice were euthanized at the same time point and histomorphometric analysis showed a corresponding decrease in tumor burden in the femora, tibiae and humeri of 2ME2 and SD-208-treated animals compared to vehicle-treated mice (Figure 13B–C). Tumor burden was further decreased by treatment with SD-208 and 2ME2 combined, compared to SD-208 alone (*P<*0.05) (Figure 13B–C). A trend towards an additional decrease with combined treatment compared to 2ME2 treatment alone did not reach significance.

We observed similar effects in a preventive model for breast cancer bone metastasis (data not shown). In this model, treatment with 2ME2 and SD-208 was initiated two days prior to tumor inoculation. Tumor burden was significantly decreased by combined treatment compared to SD-208 alone, with a trend towards an additional decrease compared to 2ME2. Together, these studies suggest that combined pharmacologic targeting of HIF-1α and TGF-β effectively reduces the development and progression of osteolytic bone metastases greater than either alone.

### Combined treatment with 2ME2 and SD-208 decreases osteoclast number at sites of bone metastases

We hypothesized that combined 2ME2 and SD-208 additionally decrease bone metastases by targeting other cells in the bone metastatic microenvironment in addition to tumor cells. Because breast cancer cells secrete factors which stimulate osteoclast formation and bone resorption in osteolytic metastases, we analyzed osteoclast number at sites of bone metastases from 2ME2- and SD-208-treated mice. Significantly fewer osteoclasts per millimeter of tumor-bone interface were present in bone metastases from 2ME2- or SD-208-treated mice compared to control mice (*P<*0.01). This number was further reduced with combined 2ME2/SD-208 treatment (*P<*0.01) (Figure 13D). These results suggest that 2ME2 and SD-208 decrease bone metastases through combined effects to reduce osteoclasts at sites of bone metastases, in addition to their actions on tumor cells.

## Discussion

Bone metastases occur in eighty percent of patients with advanced breast cancer. They are incurable and cause significant morbidity, including pain, pathologic fractures, hypercalcemia, and nerve compression syndromes due to tumor-induced osteoclastic bone resorption [1]. Standard bisphosphonate therapies improve skeletal morbidity by reducing this osteolysis [44], [45], but do not cause regression of established bone metastases. The bone microenvironment by its unique composition of growth factors housed in the mineralized bone matrix, bone resorbing and bone forming cells, promotes a feed-forward cycle of site-specific metastasis through high concentrations of growth factors, such as TGF-β [2], and local hypoxia [46]. Active TGF-β in bone [2] promotes bone metastases by increasing tumor production of factors that stimulate osteoclastic bone resorption and tumor growth [6], [7]. TGF-β-regulated factors, such as CTGF, IL-11, CXCR4 [13], [26], and MMP-1 [24], [25] are involved in multiple steps of the metastatic cascade, including invasion, homing, angiogenesis, and osteolysis [5] and constitute a gene signature for tumors that metastasize preferentially to bone [8] (Table 1). The bone microenvironment is also hypoxic [19]. Hypoxia activates signaling through HIF-1α which, like TGF-β, increases many of the factors that promote the feed-forward metastatic cycle. Although previous studies have shown that both HIF-1α and TGF-β signaling pathways are important in bone metastases, interactions between these factors have not been reported. The aim of these studies was to determine whether TGFβ and hypoxia act synergistically or work redundantly to promote bone metastases.

We first investigated interactions between the pathways *in vitro* by analyzing changes in gene expression in MDA-MB-231 breast cancer cells treated with TGF-β and 1% O_2_. Of 16 candidate genes, only two were increased by TGF-β and hypoxia: VEGF and CXCR4. VEGF, but not CXCR4, mRNA was additively increased by combined treatment with TGF-β and 1% O_2_, and promoter activation of the two factors was also additively increased by combined treatment. Previous studies of VEGF in mouse macrophages showed increased promoter activity in response to TGF-β and hypoxia and to overexpression of HIF-1α/β and Smads3/4 *in vitro* [47]. Elements responsible for TGF-β and hypoxia response were localized in the proximal region of the mouse VEGF promoter and homolog sites were identified in the human VEGF promoter [48]. The study demonstrated that TGF-β and hypoxia signaling directly crosstalk to regulate the expression of VEGF in macrophages [47]. The data here suggest that VEGF is regulated similarly by TGF-β and hypoxia in human MDA-MB-231 cells. An HRE located 1.3 kb from the transcription start site in the human CXCR4 promoter was found to mediate its response to hypoxia. Mutation of either of two putative SBEs did not significantly inhibit TGF-β-stimulated CXCR4 promoter activation. The results suggest that TGF-β regulates CXCR4 through other SBEs in the promoter that were not tested here.

MDA-MB-231 cells did not show significant additive responses to TGF-β and hypoxia for the other 14 genes examined, but other targets could be responsive. Therefore we used a comprehensive approach to genetically approach to inhibit the pathways in the tumor cells by expression of HIF-1α shRNA and a dominant-negative TβRII, which then were analyzed an *in vivo* bone metastasis model. This approach permits assessment of the tumor autonomous effects of hypoxia and TGF-β, separate from their roles in the metastatic microenvironment. Knockdown of HIF-1α mRNA in MDA-MB-231 breast cancer cells decreased osteolytic lesion area and improved survival of mice *in vivo*. Knockdown of HIF-1α did not yield a corresponding decrease in tumor burden at time of death. Tumor burden in mice euthanized at the same time point could not be compared in this survival experiment as mice bearing shHIF bone metastases lived significantly longer than the control groups. It is possible that knockdown of HIF-1α decreases osteolytic lesion development without affecting the rate of tumor growth in bone. However, this is unlikely as previous studies demonstrated that inhibition of hypoxic signaling by a dominant-negative HIF-1α decreased osteolytic bone destruction and tumor burden in mice euthanized at the same time point, while a constitutively active HIF had the opposite effect [46]. Knockdown of HIF-1α had no effect on cell proliferation *in vitro*, but it decreased primary tumor take and growth in the mammary fat pad in our experiments. Consistent with our results, Liao *et al.* reported that conditional deletion of HIF-1α from the mammary epithelium in transgenic mice delayed onset of spontaneous breast tumors and retarded their growth [49].

Together, our *in vitro* and *in vivo* results suggest that HIF-1α promotes bone metastases by regulating factors such as CXCR4, which promotes tumor cell homing to bone [50] and VEGF, which promotes angiogenesis. HIF-1α knockdown in MDA-MB-231 cells decreased CXCR4 expression *in vitro* and inhibited bone metastasis *in vivo*, with no difference in metastasis to other organs, such as the lungs or adrenal glands. The results suggest that HIF-1α knockdown may specifically inhibit skeletal metastases by blocking CXCR4-mediated homing to bone [51]. CXCR4 is more highly expressed in human breast cancer metastases to bone than to the lungs or brain [52]. Previous studies showed that treatment with CXCR4 antagonists inhibited breast cancer metastasis to bone and lung [53], [54] and decreased bone destruction due to myeloma bone metastases [55] in mice, which supports our results.

In addition, our results suggest a role for HIF-1α to regulate interactions between tumor cells and other cells in the bone microenvironment, such as endothelial cells. Knockdown of HIF-1α in MDA-MB-231 cells decreased VEGF expression *in vitro*, and inhibited tumor angiogenesis at sites of bone metastasis *in vivo*, as demonstrated by CD31 staining for endothelial cells. There was no difference in the number of vessels in shHIF mammary fat pad tumors compared to parental and shNT control tumors (data not shown). The results suggest that knockdown of HIF-1α specifically inhibits vessel formation within the hypoxic bone microenvironment, which may contribute to decreased bone metastasis in the mice. Consistent with this, treatment with a VEGF neutralizing antibody decreased tumor angiogenesis and osteolytic bone metastases in rats [56], [57].

Inhibition of either HIF-1α or TGF-β signaling in the tumor cells by shRNA knockdown or expression of a dominant-negative TβRII [6] decreased osteolytic lesion area and increased survival of mice with bone metastases compared to those bearing control cells. However, there was no additional survival benefit or reduction in lesion area with combined inhibition of these pathways. The results suggest that the two signaling pathways function in parallel and independently of one another in tumor cells. This conclusion is supported by the results *in vitro* where HIF-1α and TGF-β regulated many of the same prometastatic factors independently, with few additive responses.

Genetic inhibition tests the role of tumor cell HIF-1α and TGF-β signaling in bone metastasis but fails to address contributions from the microenvironment. In addition, shRNA knockdowns and dominant-negative receptors are not readily translatable to the clinic. Therefore we used small molecule inhibitors to inhibit these pathways systemically. 2ME2 is a naturally occurring, poorly-estrogenic metabolite of estradiol with anti-HIF, anti-angiogenic, and anti-microtubule properties [32], [58], [59]. The drug decreased osteolytic lesion area in a 4T1 mouse model of bone metastasis [42]. In our studies a soluble formulation of 2ME2 effectively inhibited HIF-1α protein expression *in vitro* for three bone metastatic cell lines: MDA-MB-231 breast, PC-3 prostate and 1205Lu melanoma cells, as demonstrated previously [32]. Systemic inhibition of HIF-1α by 2ME2 significantly decreased osteolytic lesion area and reduced tumor burden in a prevention model of MDA-MB-231 breast cancer bone metastasis, consistent with the previous studies using 4T1 cells [42]. Staining for HIF-1α and tumor hypoxia were decreased in bone metastases sections from 2ME2-treated animals, demonstrating on-target effects of 2ME2 in tumor cells *in vivo*.

Similarly, we showed that a TβRI kinase inhibitor, SD-208, significantly reduced osteolytic lesion area and decreased tumor burden in mice, while increasing survival in a dose dependent manner. SD-208 was previously shown to increase survival following orthotopic implantation of glioma cells [31]. Inhibition of TGF-β by another TβRI kinase inhibitor decreased breast cancer metastases to lungs and skeleton in mice [15].

Combined treatment with 2ME2 and SD-208 significantly decreased osteolytic lesion area on x-ray and reduced tumor burden by quantitative histomorphometry compared to vehicle or either drug alone in a clinically relevant therapeutic, as well as a prevention model of bone metastasis. Unlike the previous genetic studies where inhibition of HIF-1α and TGF-β in tumor cells had no additional effect, combined pharmacologic inhibition of these pathways with 2ME2 and SD-208 provided added therapeutic benefit, which may be due actions of the drugs on tumor cells and other cells in the bone microenvironment, such as osteoclasts. In the bone metastasis model, treatment with 2ME2 or SD-208 alone decreased the number of osteoclasts at the tumor-bone interface, which was further reduced with combined treatment. These data, together with the additive effect of 2ME2 and SD-208 on radiographic bone destruction induced by MDA-MB-231 cells, suggest that these drugs may prevent tumor-induced bone destruction by inhibiting osteoclast formation.

Systemic TGF-β blockade with SD-208 was previously shown to have profound effects on normal bone remodeling to increase bone mass in part by inhibiting osteoclast formation and bone resorption, as well as to stimulate osteoblast activity and new bone formation [60]. Here we show that 2ME2 also has direct effects on bone to increase bone mass by decreasing osteoclasts and increasing osteoblasts. 2ME2 is an inhibitor of HIF-1α, but the effects of HIF-1α in bone have been shown to be complex. Mice with a conditional deletion of HIF-1α in osteoblasts had smaller, less vascularized bones with decreased bone density [61]. In contrast, partial HIF-1α deficiency in mice heterozygous for HIF-1α prevented osteoblast apoptosis and enhanced bone mineralization and fracture repair [62]. Our results are consistent with the latter study in that 2ME2 inhibits HIF-1α but increases bone mass. In addition, HIF-1α also regulates osteoclast formation and bone resorption [63], [64] by increasing VEGF expression which substitutes for M-CSF to promote osteoclastogenesis together with RANKL [65], [66]. 2ME2 may therefore inhibit osteoclast formation and activity indirectly by blocking HIF-1α activity and VEGF secretion by osteoblasts. 2ME2 has also been shown to induce apoptosis in mature osteoclasts [42], and may have other effects in bone, as we show here. Importantly, we observed no deleterious effects of these drug treatments on the bones of animals. Unlike most current cancer therapies, including aromatase inhibitors, which cause bone loss [67], 2ME2 and SD-208 have bone-sparing effects that may contribute the beneficial effect on bone metastases [60], [68].

Our data establish that hypoxia and TGF-β signaling pathways regulate tumor-secreted factors such as CXCR4 which promotes tumor cell homing to the bone [50], and VEGF which stimulates tumor angiogenesis and increases both osteoclast [65], [69] and osteoblast activity [61]. Genetic inhibition of either HIF-1α or TGF-β in tumor cells provides proof of principle that these signaling pathways promote bone metastasis through tumor-autonomous effects. Systemic inhibition with 2ME2 or SD-208 also decreased bone metastases, while combined treatment provided additional benefit through effects on the tumor cells, as well as the bone microenvironment. Thus, combination therapy with inhibitors of hypoxia and TGF-β may significantly improve treatment and impact survival of patients with bone metastases, and provide a welcome addition to current armamentarium for bone metastases.

## Materials and Methods

### Ethics statement

Animal protocols were approved by the Institution Animal Care and Use Committee at the University of Virginia and were in accordance with guidelines from the U.S. Public Health Service Policy on Humane Care and Use of Laboratory Animals and in compliance with the U.S. Animal Welfare Act. Female athymic nude mice, 4 weeks of age, were housed under barrier conditions in laminar flow isolated hoods. Autoclaved water and mouse chow were provided ad libitum. Animals bearing human tumor xenografts were carefully monitored for established signs of distress and discomfort and were humanely euthanized when these were confirmed.

### Materials

Recombinant human TGF-β1 was purchased from R&D Systems (Minneapolis, MN). 2-methoxyestradiol (2ME2) was a gift from Entremed (Rockville, MD) and was provided as an orally-active, nanocrystalline dispersion [43]. SD-208 was obtained from Scios, Inc. (Fremont, CA) and Epichem (Murdoch, Australia). SD-208 is a potent inhibitor of TβRI (EC_50_ = 48 nM) whose selectivity profile for a variety of kinases has been previously described [31]. The drug was resuspended in 1% methylcellulose and stored at 4°C.

### Cell lines

MDA-MB-231 breast cancer cells [6] were cultured in Dulbecco's modified Eagle's media (DMEM) (Hyclone Laboratories, Logan, UT) containing 10% heat-inactivated fetal bovine serum (FBS) (Hyclone). HepG2 hepatocarcinoma cells (HB-8065, ATCC, Manassas, VA) were cultured in modified Eagle's media (MEM) supplemented with 10% FBS, sodium pyruvate and non-essential amino acids. PC-3 prostate cancer cells (CRL-1435, ATCC) were cultured in RPMI with 10% FBS. 1205Lu melanoma cells were from Dr. Alain Mauviel, Institut National de la Sante et de la Recherche Medicale, Paris, France [13]. The cells were grown in a composite medium (W489) consisting of three parts of MCDB153 and one part of L15 supplemented with 4% FBS. Cells were maintained at 37°C with 5% CO_2_ in a humidified chamber. Hypoxia treatments were performed by placing tissue culture flasks in a modular incubator chamber (model MC-101, Billups-Rothenberg Inc., Del Mar, CA), flushed with premixed 94% N_2_, 5% CO_2_, 1% O_2_ and maintained in a conventional tissue culture incubator.

### Preparation of stable clones

Parental MDA-MB-231 cells were transfected with a pLKO.1 plasmid coding an shRNA against HIF-1α (TRCNo. TRCN0000003810, MISSION® shRNA, Sigma, St. Louis, MO) using FuGENE HD (Roche, Nutley, NJ). Cells transfected with a non-target shRNA control vector (SHC002, MISSION® shRNA, Sigma) were used as a control. Single clones were selected by limiting dilution in the presence of puromycin (Invitrogen, San Diego, CA). Knockdown of HIF-1α mRNA and protein were confirmed by semi-quantitative RT-PCR and Western blot analysis. Clones were retested for stability (>80% knockdown) after culture in the absence of puromycin for 60 days. Two non-target controls (shNT) and two HIF-1α knockdown (shHIF) clones were selected for further study.

The MDA-MB-231 clonal line, MDA/TβRIIΔcyt, which stably expresses a cytoplasmically truncated type II TGF-β receptor [6], here referred to as dominant-negative receptor II (DNRII), was transfected to express either non-target or HIF-1α shRNA. Single cell clones were isolated, selected for resistance to G418 (Alexis Biochemicals, San Diego, CA) and puromycin, and tested for stable knockdown of HIF-1α as described previously. Stable DNRII expression and blockade of TGF-β signaling were confirmed by Western blot for phosphorylated Smad2. Two DNRII/ shNT and two DNRII/shHIF clones were selected for *in vivo* and *in vitro* experiments.

### MTT assay

Cell proliferation was assayed by MTT assay. Cells were plated at a density of 1000 cells/well in 96-well plates. MTT reagent (20 µl, 5 mg/ml) (Calbiochem, San Diego, CA) was added to each well. After a 5 h-incubation at 37°C, 100 µL of 0.01M HCL containing 10% SDS were added to lyse the cells and the plate was incubated at 37°C for an additional 16 h. Absorbance was measured at 570 nm using a Synergy™ HT spectrophotometer (Bio-tek, Winooski, VT).

### Semi-quantitative RT-PCR

MDA-MB-231 cells were seeded in 24-well plates (10^5^ cells/well). Forty-eight hours later, cells were starved overnight in basal DMEM media, then treated ± TGF-β1 (5 ng/mL) in DMEM-FBS (1%) and cultured in 20% or 1% O_2_ for 24 h. Cells were rinsed in PBS and then lysed in Trizol (Invitrogen) for RNA extraction. Briefly, chloroform was added to cell lysates. Samples were centrifuged (12,000 g, 15 min, 4°C) and the upper aqueous phase was collected. One volume of 70% ethanol was added, then sample was loaded on an RNeasy mini spin column (Qiagen, Valencia, CA) and total RNA was isolated according to manufacturer's instructions. DNase I treatment was performed to remove genomic DNA contamination, and RNA integrity was assessed on agarose gels. RNA (500 ng per sample) was reverse transcribed using Superscript II (Invitrogen) according to the manufacturer's instructions with anchored oligo(dT) (Abgene, Rochester, NY) for priming. The resulting cDNAs were prepared for semi-quantitative real-time PCR using QuantiTect SYBR Green PCR Kit (Qiagen) and analyzed in a MyiQ™ Single-Color Real-Time PCR Detection System (BioRad, Hercules, CA) for 40 cycles (95°C for 1 min, 58°C or 60°C for 30 s, 72°C for 30 s) after an initial 15-min incubation at 95°C. Primers were optimized for real-time PCR (amplification efficiency 100 ± 5%). Primer sequences are listed in Table S1. Target gene expression was normalized against the housekeeping gene for the ribosomal protein L32, and data were analyzed using the ΔΔCt method.

### Western blot analysis

MDA-MB-231 cells were plated in 12-well plates (2×10^5^ cells/well). Forty-eight hours later, cells were serum starved overnight in basal DMEM, then cultured in DMEM-FBS (1%) for duration of treatment. Hypoxia treatments were performed by culturing in 1%O_2_ for 6 h. TGF-β1 (5 ng/mL) treatment was for 2 h. Cells were washed once with PBS, lysed in 200 µl SDS-loading buffer, and heated to 95°C for 5 min. Samples were loaded onto a 10% polyacrylamide gel and electrophoresis was performed using a Mini Trans-Blot® cell (BioRad, Hercules, CA). Proteins were transferred onto a Hybond™-P membrane (GE Healthcare, Waukesha, WI) using a Mini-PROTEAN® Cell transfer system (BioRad). Membranes were blocked in TBS-T-5% milk for 1 h, incubated overnight with the primary antibody and for 1 h with the secondary antibody. Antibody detection was performed using Immobilon™ Western Chemiluminescent HRP Substrate (Millipore, Billerica, MA) according to the manufacturer's directions and signal was visualized on radiographic film. Antibodies used include HIF-1α (BD Biosciences, San Jose, CA), phospho-Smad2 (Ser465/467) and Smad2 (Cell Signaling, Danvers, MA); α-tubulin (Sigma) was used as a control. Anti-mouse IgG (Fab specific) and anti-rabbit IgG secondary antibodies conjugated to peroxidase were purchased from Sigma.

### Dual-luciferase assays

Cells were transfected with pGL3-luciferase constructs containing either the (CAGA)_9_ [70], VEGF or CXCR4 promoter using FuGENE HD. (CAGA)_9_ contains nine tandemly-repeated Smad binding elements. The 2.6 kb human CXCR4 promoter was from Dr. Robert Strieter, University of Virginia [40], [41], and the 3.3 kb human VEGF promoter was from Dr. Lee Ellis, University of Texas, MD Anderson Cancer Center [39]. Cells were also transfected with a phRL-renilla plasmid (Promega, Madison, WI) for normalization. Twenty-four hours later, cells were cultured serum-starved in basal DMEM medium for 4 h, then treated in the presence or absence of TGF-β1 (5 ng/mL) and 1% O_2_ for 24 h. Cells were washed once with PBS, lysed using Passive Lysis Buffer (Promega), and analyzed for luciferase activity using the Dual-Luciferase Reporter Assay System (Promega), according to the manufacturer's instructions on a FB12 Sirius luminometer (Berthold Detection Systems, Oak Ridge, TN).

### Plasmids

pCEP4-HIF-1α (MBA-2) was purchased from the ATCC; pCMV-Smad2 and -Smad3 were from Dr. David Wotton (University of Virginia); pCMV-Smad4 was from Dr. Rik Derynk (University of California San Francisco). VEGF and CXCR4 promoter deletion mutants were generated using forward primers containing a 5′ KpnI restriction site and 3′ end complementary to the promoter (Table S2). Reverse primer (5′-GTTCCATCTTCCAGCGGATA-3′) binds a region of the luciferase coding sequence. Promoter fragments were amplified by PCR using PfuUltra™ Hotstart DNA polymerase (Stratagene, La Jolla, CA). Products were digested overnight with KpnI and XhoI, purified on agarose gel (QIAquick Gel Extraction kit, Qiagen), and ligated into the pGL3-luciferase vector using T4 DNA ligase (Roche) according to the manufacturer's instructions. QuikChange® II Site-Directed Mutagenesis kit (Stratagene) was used to mutate putative Smad-binding (SBE) and hypoxia response (HRE) elements in the VEGF and CXCR4 promoters. Primers were designed using the QuikChange® Primer Design Program (Stratagene) and mutagenesis performed according to the manufacturer's protocol.

### VEGF enzyme-linked immunosorbant assays

MDA-MB-231 parental cells and clones were plated in 12-well plates (2×10^5^ cells/well) and grown for 48 h. After 4 h serum starvation, cells were treated ± TGF-β1 (5 ng/mL) in DMEM-FBS (1%) and ± 1% O_2_ for 24 h. Conditioned media was collected and analyzed by ELISA assay for VEGF-A (Bender MedSystems, Inc., Burlingame, CA), according to the manufacturer's instructions. Cell number per well was used for normalization.

### Flow cytometry

MDA-MB-231 parental cells and clones were plated in triplicate in 6-well plates (5×10^5^ cells/well). Forty-eight hours later, cells were trypsinized, counted, and 5×10^5^ cells per sample were transferred to 5 mL tubes. Cells were centrifuged (1,000 rpm, 5 min, 4°C), washed twice with flow cytometry buffer (FCB) (2% bovine serum albumin and 0.2% sodium azide in PBS 1X) and incubated with phycoerythrin-conjugated CXCR4 antibody (eBiosciences, San Diego, CA) for 30 min. Cells were washed twice, fixed in paraformaldehyde (2% in PBS 1X, 15 min, RT) and resuspended in FCB. A FACSCalibur flow cytometer (Becton Dickinson, Franklin Lakes, NJ) was used for flow cytometry, followed by analysis with FloJo software (TreeStar, Ashland OR).

### In vivo protocols

#### Bone metastasis model

Intracardiac inoculation of tumor cells was performed as previously described [71]. Tumor cells were trypsinized, washed twice and resuspended in PBS to a final concentration of 10^5^ cells in 100 µl. Animals were anesthetized with ketamine/xylazine and positioned ventral side up. MDA-MB-231 parental or clonally-derived cells (n = 12–15) were inoculated into the left ventricle by percutaneous injection using a 26-gauge needle.

#### Mammary fat pad tumor model

Four week old female nude mice were anaesthetized with ketamine/xylazine and placed in supine position. MDA-MB-231 parental or clonally-derived cells (10^6^ cells in 100 µL PBS; n = 10 mice per cell line) were inoculated into the upper mammary fat pad using a 27-gauge needle. Tumor size was followed by measuring tumor diameters with calipers three times per week and tumor volume was calculated by the formula of an ovoid: tumor volume = 4/3π×*L*/2 (*w*/2)^2^, where *L* and *w* equal mid-axis length and width, respectively.

#### Drug treatments

2ME2 (150 mg/kg) or its vehicle PBS was administered daily by i.p. injection. In the first experiment, SD-208 was administered preventively via the drinking water (either 0.3 or 1 mg/mL) beginning two days prior to tumor inoculation. Vehicle control animals were given water without compound. In all subsequent experiments, SD-208 (60 mg/kg) or its vehicle 1% methylcellulose was administered daily by oral gavage. In the preventive protocols, drug treatment was initiated two days prior to tumor cell inoculation and continued daily for the duration of the study. In the therapeutic protocol, drug treatment was initiated when osteolysis was observed on x-ray and then continued during the duration of the study.

#### Radiography

Osteolytic lesions were analyzed by radiography using a Faxitron MX-20 with digital camera (Faxitron X-ray Corporation, Wheeling, IL). Mice were imaged in a prone position at 1×magnification and 4× when osteolytic lesions were suspected. Osteolytic lesion area was quantified using MetaMorph software (Universal Imaging Corporation, Dowingtown, PA).

#### Bone mineral density (BMD) measurement

BMD was performed on live mice using a GE Lunar PIXImus II (GE Healthcare) mouse densitometer (Faxitron X-ray Corporation). Measurements were performed one time/week throughout the experiment. The densitometer was calibrated with a plastic embedded murine phantom before use. Mice were anesthetized, placed on an adhesive tray in a prone position with limbs spread. Total body measurement was performed excluding the calvarium, mandible and teeth. A region of interest was defined at the distal femur, proximal tibia just beneath the growth plate (12×12 pixels) and the lower lumber spine (20×50 pixels). Values were expressed as percentage change in BMD over base line in mg/cm^2^.

#### Bone histology & histomorphometry

Forelimbs, hindlimbs, and spine of the mice were collected upon euthanasia and fixed in 10% neutral buffered formalin for 48 h and decalcified in 10% EDTA for 2 weeks. After decalcification tissues were processed in a Shandon Excelsior automated tissue processor (Thermo Fisher Scientific, Waltham, MA) and embedded in paraffin wax for sectioning. Longitudinal, mid-sagittal sections 3.5 µm in thickness from the tibia, femur and lumbar spines were cut using an automated Microm HM 355 S microtome (Thermo Fisher Scientific). Tissue sections were stained with hematoxylin and eosin (H&E) and prepared for histomorphometric analysis. All sections were viewed on a Leica DM LB compound microscope (Leica Microsystems, Bannockburn, IL) with a Q-Imaging Micropublisher Cooled CCD color digital camera (Vashaw Scientific Inc., Washington, DC). Images were captured and analyzed using MetaMorph software (Universal Imaging Corporation). Tumor burden per bone, defined as area of bone occupied by the cancer cells, was calculated at the tibia, femur and humerus at 50×magnification on H&E stained sections, as previously described [72]. Osteoclast number at the tumor-bone interface (OC/mm bone surface) in the femur, tibia and humerus was measured on TRAP stained slides at 200×magnification. For normal bone, osteoblast (OB) number and osteoclast (OCL) number at the bone surface were measured in the distal femur and proximal tibia at 200×magnification on H&E and TRAP stained slides, respectively.

#### Hypoxyprobe^ TM^-1 staining for tumor hypoxia

For assessment of tumor hypoxia, mice were injected 2 h prior to euthanasia with pimonidazole (60 mg/kg i.p.) and sections stained with Hypoxyprobe^TM^-1 kit (Natural Pharmacia International, Inc., Burlington, MA) according to the manufacturer's instructions. Tumor hypoxia in bone metastases tumor sections was scored semi-quantitatively on a 1–4 + scale, based on the percentage of positively stained tumor within a 400× field: grade 1: 25% staining, 2: 50%, 3: 75%, and 4: 100%.

#### Immunohistochemistry

Immunohistochemical analysis was performed on decalcified paraffin-embedded tissue sections. Antibodies against HIF-1α and CD31 were purchased from BD Biosciences. All staining was performed using VECTASTAIN® Elite ABC kit (Vector Laboratories, Burlingame, CA). Slides were stained using a 3,3, diaminobenzidine substrate kit (Vector Laboratories) and counterstained with hematoxylin. HIF-1α and CD31 staining were quantified by the percentage of positively staining nuclei per 400× field and number of vessels per 200× field, respectively. Three or more fields per animal were analyzed and averaged. Averages for 3 or more animals per group were compared.

#### TUNEL assay

Tumor cell apoptosis was analyzed in bone metastases tissue sections using the DeadEnd^TM^ Colorimetric TUNEL system (Promega), according to the manufacturer's instructions.

### Statistical Analyses

#### In Vivo

All data were analyzed with the use of Graphpad Prism v4.0 software (GraphPad Software, Inc., La Jolla, CA). Differences in osteolytic lesion area between clones and treatment groups were analyzed by two-way ANOVA. Histomorphometry for tumor burden and osteoclast number was analyzed by one-way ANOVA and Tukey's or Newman-Keuls multiple comparison test. Kaplan-Meier survival curve data was analyzed by a Logrank test. All the results were expressed as mean ± SEM, and *p<*0.05 was considered significant.

#### In Vitro

All data were analyzed with the use of Graphpad Prism v4.0 software (GraphPad Software, Inc., La Jolla, CA). Samples were analyzed in triplicate for RT-PCR, dual-luciferase assays, ELISA, and flow cytometry and statistics analyzed by unpaired t-test. Results are expressed as mean ± standard deviation, and *p<*0.05 was considered significant. Immunostaining was analyzed by one-way ANOVA or unpaired t-test.

## Supporting Information

Table S1 Sequences of primers for human genes analyzed by semi-quantitative RT-PCR.
(0.04 MB DOC)

Table S2 PCR primer sequences for 5′ to 3′ deletion of VEGF and CXCR4 promoters.
(0.03 MB DOC)
