# Supplementary material for: Hypoxia and TGF-β Drive Breast Cancer Bone Metastases through Parallel Signaling Pathways in Tumor Cells and the Bone Microenvironment
Source: PLoS One. 2009 Sep 3;4(9):e6896. doi: 10.1371/journal.pone.0006896 (PMC2731927; doi:10.1371/journal.pone.0006896)
Supplement: Table S1 — Sequences of primers for human genes analyzed by semi-quantitative RT-PCR. (0.04 MB DOC) [file pone.0006896.s001.doc]

**Table S1. Sequences of primers for human genes analyzed by semi-quantitative RT-PCR.**

| **RT-PCR Primer** | **GeneID** | **Sense (5’🡪3’)** | **Antisense (5’🡪3’)** |
| --- | --- | --- | --- |
| CTGF | 1490 | GCTACCACATTTCCTACCTAGAAATCA | GACAGTCCGTCAAAACAGATTGTT |
| CXCR4 | 7852 | CCGTGGCAAACTGGTACTTT | GACGCCAACATAGACCACCT |
| HIF-1α | 3091 | CACAGAAATGGCCTTGTGAA | CCAAGCAGGTCATAGGTGGT |
| IL-6 | 3569 | GAAAGCAGCAAAGAGGCACT | TTTCACCAGGCAAGTCTCCT |
| IL-8 | 3576 | ACTGAGAGTGATTGAGAGTGGAC | AACCCTCTGCACCCAGTTTTC |
| IL-11 | 3589 | TGAAGACTCGGCTGTGACC | CCTCACGGAAGGACTGTCTC |
| PHD2 | 54583 | AATCTGGGAGCCTGATTCCT | GTGGCTATTGCGATCCTCAT |
| PTHrP | 5744 | ACTCGCTCTGCCTGGTTAGA | GGAGGTGTCAGACAGGTGGT |
| RPL32 | 6161 | TCAGGTGATCTTCCCACCTC | ACCACATCCCATATCCCTCA |
| Ski | 6497 | CAGGAGCTGGAGTTCCTACG | GTGACTCGTTGGCCTCTTTC |
| Smad2 | 4087 | GGAATTTGCTGCTCTTCTGG | TCTGCCTTCGGTATTCTGCT |
| Smad3 | 4088 | CATAGGTGCTTTGGGCGTAT | CTGCTATCCAGTCACCAGCA |
| Smad4 | 4089 | TTGGGGCCCTTAACCTTATC | AGCCATGCCTGACAAGTTCT |
| Smad7 | 4092 | CCAACTGCAGACTGTCCAGA | CAGGCTCCAGAAGAAGTTGG |
| SnoN | 6498 | TGCCCCAAATGTGTCACTTA | TCCATTTTCTCCTGTTCCTCA |
| TGF-β1 | 7040 | AGGACTGCGGATCTCTGTGT | GGGCAAAGGAATAGTGCAGA |
| VEGF | 7422 | AAGGAGGAGGGCAGAATCAT | CACACAGGATGGCTTGAAGA |
