# Supplementary material for: Hypoxia and TGF-β Drive Breast Cancer Bone Metastases through Parallel Signaling Pathways in Tumor Cells and the Bone Microenvironment
Source: PLoS One. 2009 Sep 3;4(9):e6896. doi: 10.1371/journal.pone.0006896 (PMC2731927; doi:10.1371/journal.pone.0006896)
Supplement: Table S2 — PCR primer sequences for 5′ to 3′ deletion of VEGF and CXCR4 promoters. (0.03 MB DOC) [file pone.0006896.s002.doc]

**Table S2. PCR primer sequences for 5’🡪3’ deletion of VEGF and CXCR4 promoters.**

| **Promoter** | **Length** | **Bases** | **Primer (5’🡪3’)** |
| --- | --- | --- | --- |
| CXCR4 | 2.2kb | -2216 to +2 | **cggggtaccccg**cttccttttagtagagatccc |
| CXCR4 | 1.0kb | -953 to +2 | **cggggtaccccg**ctccgggcttatttgctgg |
| VEGF | 2.1kb | -1187 to +957 | **cggggtaccccg**gctctgggcagctggcc |
| VEGF | 1.8kb | -843 to +957 | **cggggtaccccg**ggaccccagtcactccag |

Bold indicates KpnI restriction site.
